# Association between sick child facility readiness and quality of care at the individual and facility level in five low- and middle-income countries

**DOI:** 10.1186/s12913-024-11772-9

**Published:** 2024-11-14

**Authors:** Emily D. Carter, Ashley Sheffel, Jennifer Requejo, Margaret Kosek, Harry Campbell, Thom Eisele, Melinda K. Munos

**Affiliations:** 1 Johns Hopkins Bloomberg School of Public Health, Baltimore, MD USA; 2 World Bank Group, Washington, DC USA; 3 University of Virginia, Charlottesville, VA USA; 4 University of Edinburgh, Edinburgh, UK; 5 Tulane School of Public Health and Tropical Medicine, New Orleans, LA USA

**Keywords:** Quality of care, Service readiness, Health services research, Child health, International health

## Abstract

**Background:**

Raising the quality of health services is key to continued progress in improving child health, however, data on service quality are limited and difficult to interpret. The relationship between facility readiness and the quality of care is complex.

**Methods:**

Using publicly available data sets from five low- and middle-income countries (LMICs), we assessed the relationship between structural factors and the clinical quality of care for managing sick children. We developed indices for readiness and quality accounting for available indicators, expert opinion, and alignment with integrated management of childhood illness (IMCI) guidelines. In each country, we assessed the association between readiness and quality, with and without adjusting for other factors. We considered associations overall, by domain, and by provider type, explored non-linear associations, and compared associations at the individual and facility-level.

**Results:**

The analysis included data from 3,149 health facilities and 11,159 sick child observations. In four of the five countries included in the analysis, we observed for every 10%-point increase in readiness, quality increased by about 1% point after adjusting for facility type and managing authority. There was little evidence of a non-linear relationship or a threshold effect altering the relationship between readiness and quality of care. Beyond readiness, younger child age, higher cost of care, and having a respiratory, digestive, or febrile diagnosis were most often associated with a higher quality of care. Higher “human resources” readiness domain scores were most consistently associated with better quality of care, while the quality of care domain of “treatment” was the least influenced by readiness. Facility-level associations did not vary greatly from individual-level associations.

**Conclusions:**

The weak correlation observed suggests readiness plays an important role in quality but as currently measured cannot be used to characterize clinical quality of care. Data for assessing quality of health services are limited, presenting challenges for understanding impediments, assessing interventions, and gauging changes in the quality of care over time. We need better data to assess the quality of care being delivered in LMICs to understand what factors drive quality, with the goal of improving the management of sick children.

**Supplementary Information:**

The online version contains supplementary material available at 10.1186/s12913-024-11772-9.

## Background

With the Sustainable Development Goals’ renewed focus on universal health coverage, there has been increasing interest in understanding and improving health care quality within the context of reproductive, maternal, newborn, and child health (RMNCH) in low- and middle-income countries (LMICs). Insufficient quality of care has been identified as a necessary area of improvement to achieve national and global health targets [1]. However, data around the quality of care available in LMICs is often limited and difficult to interpret.

Defining and measuring quality of health care can be complex. Numerous health care quality frameworks exist [1–3]. Common across the frameworks, each incorporates some aspect of structural factors (e.g., amenities, commodities, workforce) impacting on the quality of care and processes of health workers delivering care and ultimately health outcomes. Numerous analyses have attempted to define these constructs using existing data [4]. In LMICs, standardized data for assessing healthcare quality are typically derived from health facility surveys such as the WHO Harmonized Health Facility Assessment [5] and World Bank Service Delivery Indicators survey [6]. These assessments collect data on health facility amenities, equipment, medicines and commodities, diagnostics, and health workforce [7]. Some surveys, such as the Service Provision Assessment (SPA) [8], also include observation of client-provider interactions for select health services and exit interviews with clients. These data provide extensive structural indicators, but more limited information related provision of health care and experience of care [7]. In analyses and characterizations of the health system, the specific indicators selected for defining the different aspects of quality vary, from concise to expansive lists of items, and narrow to broad definitions of the constructs [4].

In conceptual models, structural factors are typically considered a prerequisite for appropriate care actions to occur, as sufficient infrastructure and commodities are required to both assess and treat a patient. However, the degree to which provision of appropriate clinical care, in the form of actions taken by the health care provider, is influenced by these factors is variable. How well the two constructs correlate, and the degree to which differences in structural quality can account for variation in process quality, is also complex. Analyses have addressed these questions as they relate to RMNCH in LMICs using varied methods and have found mixed results [4]. Numerous publications have assessed the influence of quality improvement strategies and patient, health worker, and facility factors on appropriate management of specific sick child conditions in different LMICs [9–16]. Two multi-country studies found limited association between structural and process quality for cutting across health service areas [17, 18], one study found a small but significant effect of structural quality limiting provision of high quality antenatal care [19].

Using publicly available SPA data sets, we set out to create robust definitions of facility readiness (structural quality) and clinical quality of care (process quality / provision of care) for management of sick children incorporating both existing guidelines and expert opinion applied to available indicators. We then assessed how these two constructs relate to each other to better understand to what degree structural factors enable or restrict the clinical quality of care. Our analysis uses a large sample of sick child observations derived from multiple countries, focuses on the appropriate management of any sick child rather than specific conditions, and attempts to isolate and describe the relationship between readiness and quality of care by adjusting for other health facility, health worker, child, caretaker, and illness episode factors.

## Methods

We developed indices of facility readiness and clinical quality of care for sick children. We then assessed the association between readiness and quality using SPA data from five countries.

### Source of readiness and quality data

We created readiness and quality indices using SPA data from Haiti, Malawi, Nepal, Senegal, and Tanzania. The SPA is administered to facility-based providers within a country and is typically sampled to produce a representative estimate of the service environment at a regional level and by facility type (e.g., first-level facility, referral hospital) and managing authority (e.g., public, private) at a national level. Some SPAs are administered to a census of health facilities, including the 2013 Haiti and 2013/2014 Malawi SPA. The SPA collects data on child health services through (1) a health facility inventory of service readiness, (2) interviews with health care providers around training and support, (3) direct observations of health care provider interactions with sick child clients, and (4) exit interviews with the caretakers of observed sick children (Table 1).

**Table 1:**
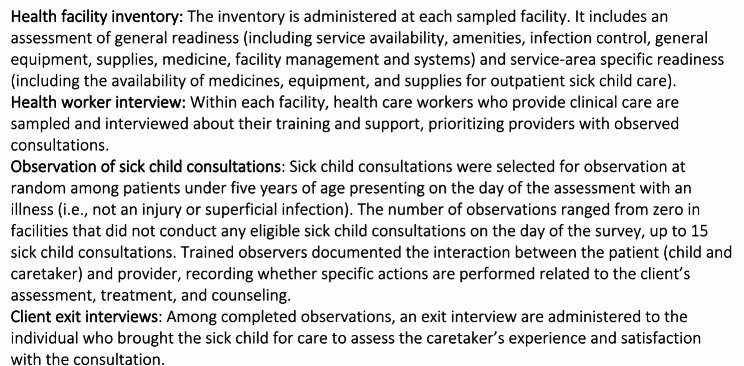
Details on SPA sampling

### Definition of indices

We extracted items from the SPA which aligned with integrated management of childhood illness (IMCI) or country-specific guidelines for the appropriate assessment, treatment (or referral), and counseling for common childhood illnesses [20]. We also included items related to integrated care administered as part of IMCI protocol (e.g., review of vaccination records). Extracted items included relevant actions performed by health workers, structural items (e.g., infrastructure or commodities) required as inputs for these actions, and interactions with the sick child’s caretaker.

We surveyed a broad group of global child health experts to select the most appropriate items to include in readiness and quality scores. To capture the perspective of individuals with expertise in clinical care in LMIC settings, we circulated the survey to the Child Health Task Force, individuals involved in the evaluation of the IMCI protocol, and experts in pneumonia, malaria, and diarrhea management in LMICs. In addition, these individuals were encouraged to circulate the survey to other colleagues with expertise in the field. We developed and administered the survey online using Qualtrics (QualtricsXM, Seattle WA), from September 1, 2020, until October 15, 2020. Out of the 40 complete survey responses received, 29 respondents reported having a clinical background. Respondents were evenly distributed across NGO, academic, and government institutions. Most respondents were based in high-income countries (*n* = 24), followed by sub-Saharan Africa (*n* = 8), and South or Southeast Asia (*n* = 6).

The survey asked experts to rate the importance of each item for the delivery of high-quality child curative services (“unimportant,” “somewhat important,” “very important,” or “essential”). Items ranked as “essential” by at least half of the respondents were included in the index. Select items with borderline rankings were added or removed from the index to ensure continuity between the readiness and quality scores and consistency with IMCI guidelines (e.g., inclusion of diazepam as treatment for severe/referral conditions). The full list of items considered and ranking of items by domain are provided in Supplementary Boxes 1 and 2. A table of the final items included in the score are presented in Tables 2 and 3. The readiness index consisted of four domains: (1) amenities (infrastructure and emergency transport), (2) equipment and supplies (infection prevention and control, diagnostic tools, equipment for treatment administration), (3) preventative and curative medicines and commodities, and (4) human resources (staff and relevant job aids). The clinical quality index included four domains aligned with the steps in the IMCI protocol: (1) assessment of the sick child (client history and physical exam), (2) treatment based on diagnosis (treatment prescribed or referral for more advanced care), (3) counselling of caretaker on management of the child, and (4) integrated care (vaccination, deworming, vitamin A supplementation, growth and feeding). In the absence of a clinical re-examination of the client or objective exam and history results, provider-reported diagnosis was the best information available for classifying the appropriateness of prescribed treatment.

**Table 2:** Readiness index

Readiness indicators	
Items by Domain	Function:
Amenities: Facility amenities	
• Primarily uses an improved water source	Safe environment
• Improved latrine available for general outpatient use	Safe environment
• Regular access to power	Safe environment
• Access to functional emergency transport	Facilitated referral capacity
Equipment: Equipment available and functional	
• Thermometer	Assess temperature
• Stethoscope	Checking breathing sounds and heart rate
• Timer	Count breathing rate
• Infant scale	Weigh infants
• Child scale	Weigh children
• Height board	Measure child height
• Pulse oximeter	Assess blood oxygen level
• Malaria test (RDT or microscopy)	Assess malaria parasitemia
• Single use syringe	Administer injectable medicines
• Cannula	Administer intravenous medicines
• Pediatric self-inflating bag and mask	Administer oxygen
• Oxygen (cylinders, concentrator, or distribution system)	Administer oxygen
• Disposable gloves	Safe patient exam
• Medical masks	Safe patient exam
• Water and soap, or alcohol rub	Safe patient exam
• Disinfectant	Safe patient exam / environment
• Sharps disposal	Safe environment
• Infectious waste disposal	Safe environment
Medicines/commodities: Medicines or commodities available and in-date	
• ORS	Management of mild/moderate dehydration
• First-line oral antimalarial	Management of uncomplicated malaria
• Oral antibiotic	Management of multiple conditions, including uncomplicated pneumonia
• Intravenous fluids (ringers’ lactate, saline, or dextrose)	Management of severe dehydration
• Injectable antibiotic (ceftriaxone, gentamicin, ampicillin or penicillin)	Management of severe pneumonia/sepsis
• Injectable or suppository antimalarial (injectable quinine or artesunate, or artesunate suppository)	Management of severe malaria
• Diazepam	Management of convulsions
• Bronchodilator	Management of asthma
• Vitamin A	Management of measles / prevent deficiency
• Paracetamol	Management of fever
• Dewormer	Management of parasites
Human resources:	
• Proportion of staff trained in sick child care in last 2 years (among those who treat children)	Sick child training
• Guidelines on sick child management	Sick child job aid

**Table 3:** Quality index

Quality	
Items by Domain	Function:
Assessment/exam: Assessment of sick child including client history and physical exam	
• Assessed history of fever	Primary symptom for IMCI algorithm
• Assessed history of cough or difficult breathing	Primary symptom for IMCI algorithm
• Assessed history of diarrhea	Primary symptom for IMCI algorithm
• Assessed history of ear pain	Primary symptom for IMCI algorithm
• Assessed history of convulsions with illness	Primary symptom for IMCI algorithm – referral
• Assessed history of severe vomiting during illness	Primary symptom for IMCI algorithm – referral
• Assessed history of child unable to drink or breastfeed during illness	Primary symptom for IMCI algorithm – referral
• Asked about feeding / breastfeeding habits during illness	Assess feeding issues during illness
• Observed whether child could drink or breastfeed	Assess inability to drink – immediate referral
• Child undressed	Examine for undernutrition or other symptoms
• Child’s temperature checked	Assess current fever
• Child weighed	Assess nutritional status
• Checked for palmar pallor	Assess anemia
• Checked for edema	Assess nutritional status
• Auscultated or counted breaths (among children with history of cough)	Assess cough, rapid or difficult breathing
• Checked for neck stiffness (among children with history of fever)	Assess meningitis
• Skin turgor assessed (among children with history of diarrhea)	Assess dehydration
• Felt behind ear (among children with history of ear pain)	Assess mastoiditis
• Malaria diagnosis based on RDT/microscopy	Test for malaria
Treatment: Treatment or referral based on diagnosis	
• Prescribed antibiotic for diagnosis of pneumonia	Treatment of pneumonia
• Prescribed antibiotic for diagnosis of typhoid	Treatment of typhoid
• Prescribed antibiotic for diagnosis of mastoiditis	Treatment of mastoiditis
• Prescribed antibiotic for diagnosis of acute ear infection	Treatment of acute ear infection
• Prescribed antibiotic for diagnosis of amebiasis	Treatment of amebiasis
• Prescribed antibiotic for diagnosis of UTI	Treatment of UTI
• Prescribed injectable antibiotic for diagnosis of meningitis	Treatment of meningitis
• Prescribed injectable antibiotic for diagnosis of septicemia	Treatment of septicemia
• Prescribed antimalarial for diagnosis of malaria	Treatment of malaria
• Prescribed IV for diagnosis of severe dehydration	Treatment of severe dehydration
• Prescribed ORS for diagnosis of mild/moderate dehydration	Treatment of mild/moderate dehydration
• Prescribed zinc for diagnosis of diarrhea	Treatment of diarrhea
• Prescribed vitamin A for diagnosis of measles	Treatment of vitamin A
• Prescribed bronchodilator for diagnosis of asthma / bronchial spasm	Treatment of asthma
• Counselled on feeding with diagnosis of mild or moderate malnutrition	Treatment of mild/moderate malnutrition
• Admitted or referred with diagnosis of severe malnutrition	Treatment of severe malnutrition
• Referral:	Appropriate referral
◦ Gave referral slip to caretaker	
◦ Explained when to go for referral	
◦ Explained the reason for referral	
◦ Explained where (or to whom) to go	
Counseling: Counseling of caretaker on management of the child	
• Told the caretaker what illness(es) the child has	Gave diagnosis
• Described signs and/or symptoms in the child for which to immediately bring child back	When to return
• Discussed follow-up visit for the sick child	When to return
• Told the caretaker to give extra fluids to the child during this illness	Appropriate feeding during illness
• Told the caretaker to continue feeding the child during this illness	Appropriate feeding during illness
• Explained how to administer oral treatment(s) to be taken at home	How to administer home treatment
• Asked the caretaker to repeat the instructions for giving medications at home	How to administer home treatment
• Caretaker reported comfortable giving medication at home	How to administer home treatment
Integrated care: Integration of preventative services and well-child care	
• Asked if child received any de-worming medication in last 6 months	Deworming
• Asked if child received Vitamin A within past 6 months	Vitamin A
• Looked at the child’s immunization card or asked caretaker about child vaccination history	Vaccination
• Mentioned the child’s weight or growth to the caretaker, or discussed growth chart	Growth
• Provided general information about feeding or breastfeeding the child even when not sick	Feeding

We adapted the included items for each country to align with country data availability and minor variations in guidelines (e.g., recommended first-line antimalarial). A score of one was assigned if an item was available or relevant action was performed or zero if an item was unavailable or relevant action was not performed. Actions were only included in the quality score for an individual sick child observation if clinically appropriate based on the available observation data (e.g., treatment with an antimalarial was only included for children with a malaria diagnosis – if no malaria diagnosis was given the associated treatment action was excluded from the score calculation). Each item received equal weight within a domain and domain scores were averaged to generate the overall readiness and quality scores resulting in domain and full index scores that ranged from 0 to 1.

We restricted the analysis to children between the ages of 2 and 59 months to align with the age group included in the IMCI sick child guidelines. We further restricted our analysis to include only those observations that ended with the child being discharged from the facility, either returning home or referred to another health facility. This ensured cases included in the analysis offered a complete picture of the care received for a discrete care-seeking event, which we could not achieve for children admitted for care, sent for lab work, or sent elsewhere within the facility and subsequently lost to observation.

### Assessing association between readiness and quality

After constructing the readiness and quality indices, we examined the distribution of facility readiness and quality scores accounting for the sample design of each SPA survey. We descriptively examined the distribution of facility-level readiness and quality by country, by provider category, and by index domain.

We next examined the association between facility readiness and the clinical quality of care delivered to individual sick children. At the individual level, we assessed if there was an association between readiness and quality (1) without adjusting for other factors, (2) adjusting for the type of health facility and managing authority group (i.e., provider category), and (3) adjusting for characteristics of the health facility, health worker, child, caretaker, and illness episode. Additional facility-level covariates included the region (district in Malawi), facility type (e.g., district hospital, health center) and managing authority (e.g., public, private, NGO) as categorized by each country survey, and whether the facility was urban or rural. Health worker covariates included health worker’s qualification (classified in descending order of years of pre-service training as doctors, medical officers, nurses, health assistants, and other) and sex. Child and caretaker factors including child age (months), child sex, caretaker age, caretaker education (none, any primary, any secondary or higher), and caretaker’s relationship to the child (mother, father, sibling, grandparent, aunt/uncle, or other). Finally, covariates specific to the care-seeking episode included the diagnosis provided by the health worker (respiratory illness, digestive illness, malaria, non-malarial febrile illness, other illness, and multiple illness categories) and the standardized cost of the visit. We used a linear mixed-effects model with the observation as the unit of analysis, with clustering at the health facility and health worker levels and assuming independent variance. As the primary objective of our analysis was to isolate the association between readiness and quality, we did not include any correction for multiple comparisons in the adjusted models. Accordingly, a portion of statistically significant associations may occur due to Type I errors.

We further examined differences in the association between readiness and quality by facility type and managing authority to assess if different categories of providers demonstrated unique relationships between their readiness and quality. To gauge a potential non-linear or threshold effect between readiness and quality, we assessed the statistical significance of marginal spline models for deviation from a linear association by (1) relative quintile of provider category readiness or (2) absolute readiness scores (e.g., above or below 50, or binned into 20 pt increments). Finally, we evaluated the association between quality and each domain of readiness (i.e., amenities, equipment, commodities, and human resources) to understand if a specific component of readiness was more strongly associated with the outcome of quality. We also examined the relationship between readiness and each quality domain (i.e., assessment, treatment, counseling, and integrated care) to assess whether specific aspects of quality were more sensitive to readiness. The domain-level associations were also adjusted for facility type and managing authority.

We repeated the analysis at the facility level, examining the association between facility readiness and quality, averaging individual observation quality scores within a facility accounting for client observation weights.

Based on the primary analysis results, we also conducted a sensitivity analysis removing the “treatment” domain from the quality score to assess whether the domain influenced the association between readiness and quality. This decision was based on the observations from our initial results including, (1) the lack of association between readiness and the quality treatment domain, (2) the fact that individual treatment domain scores were often missing or heavily skewed to either 0 or 1, and (3) the fact that treatment indicators were necessarily conditioned on an unconfirmed, provider-reported diagnosis.

## Results

### Distribution of scores by survey

The sample size varied greatly by country, with the smallest number of facilities that provide curative services for children and the sample of sick child / health worker interactions observed in Senegal and the greatest in Tanzania (Table 4). In Malawi and Haiti, the SPA included a census of health facilities offering child curative services. Between 10% (Malawi) to 42% (Haiti) of sick child observations were excluded because the child was either admitted or sent elsewhere in the facility for care (e.g., lab), preventing complete observation of the care received. In total, we included data from 3,149 health facilities and 11,159 sick child observations in the analysis.

**Table 4:** Description of Service Provision assessments (SPAs) included in the analysis

Country	Survey Year	Number of facilities that treat sick children	Number of facilities with at least one sick child observation meeting inclusion criteria	Number of sick child observations	Number of sick child observations meeting inclusion criteria
Haiti	2013	848	515	2450	1422
Malawi	2013/2014	920	739	3437	3083
Nepal	2015	907	654	2229	1866
Senegal	2012/2013	413	320	1307	1075
Tanzania	2014/2015	1154	921	4959	3713

**Fig. 1:**
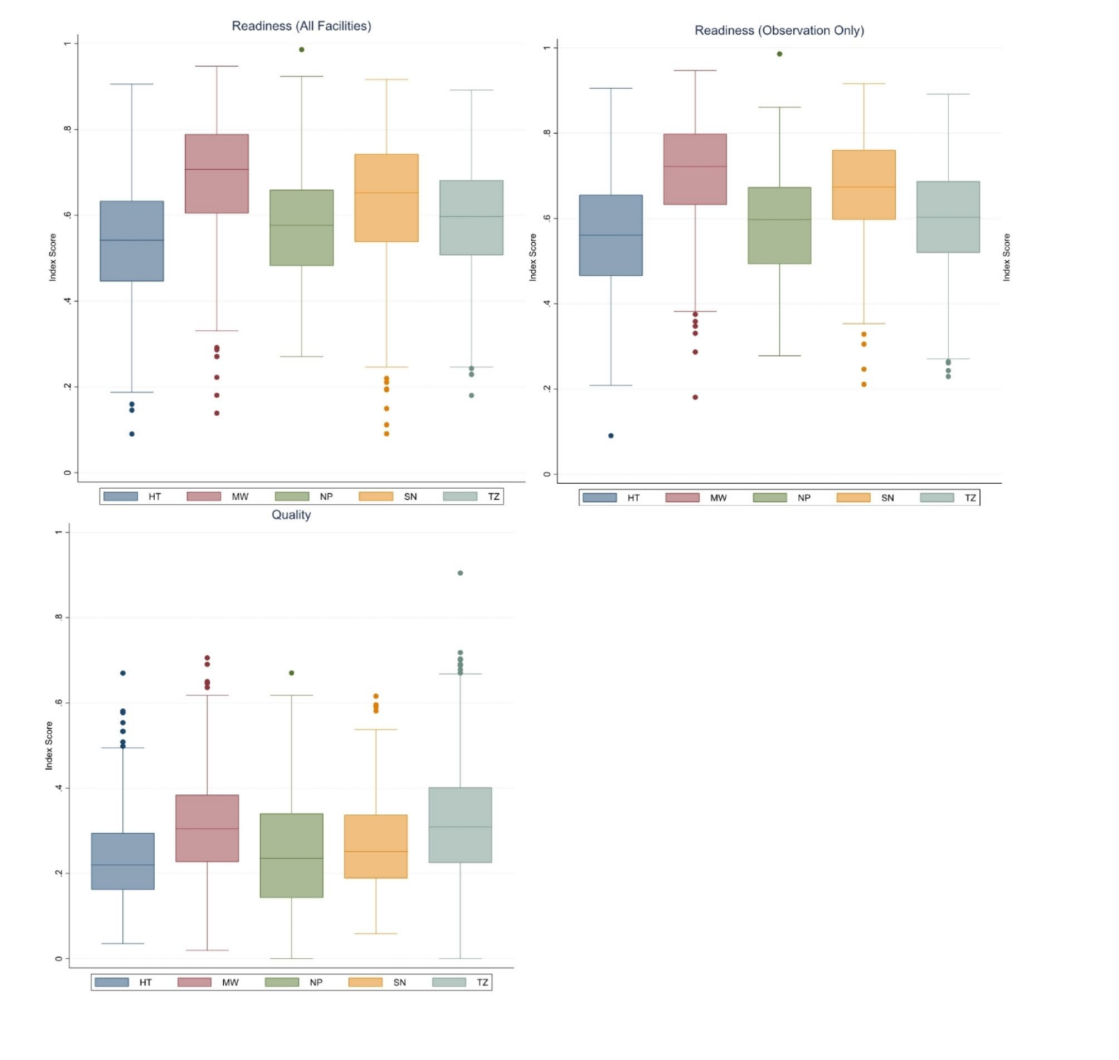
Distribution of (**a**) readiness scores among all facilities, (**b**) readiness among facilities with observations of sick children, and (**c**) facility-level quality by country

**Fig. 2:**
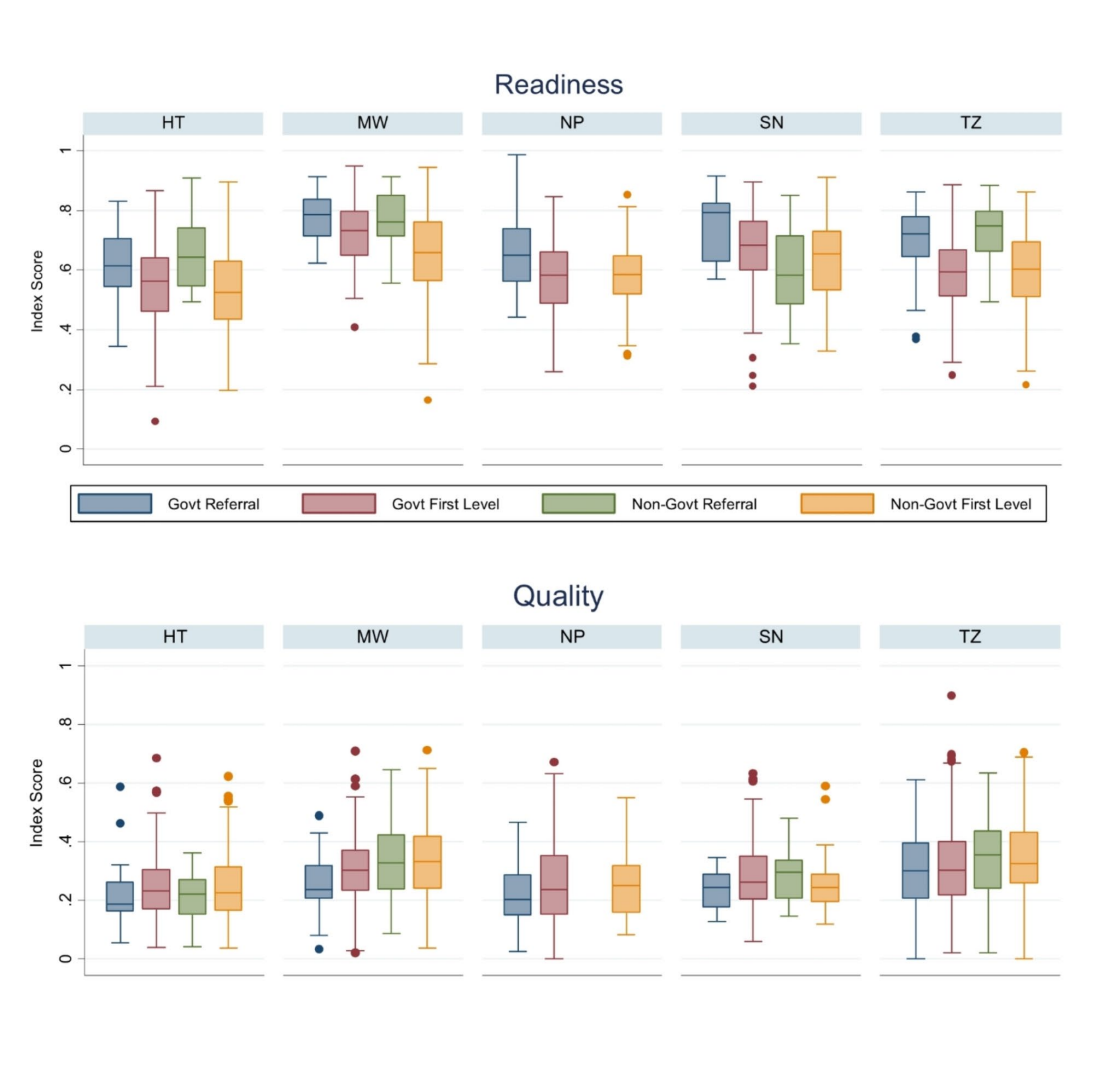
Distribution in facility-level (**a**) readiness and (**b**) quality, by provider category and country

**Fig. 3:**
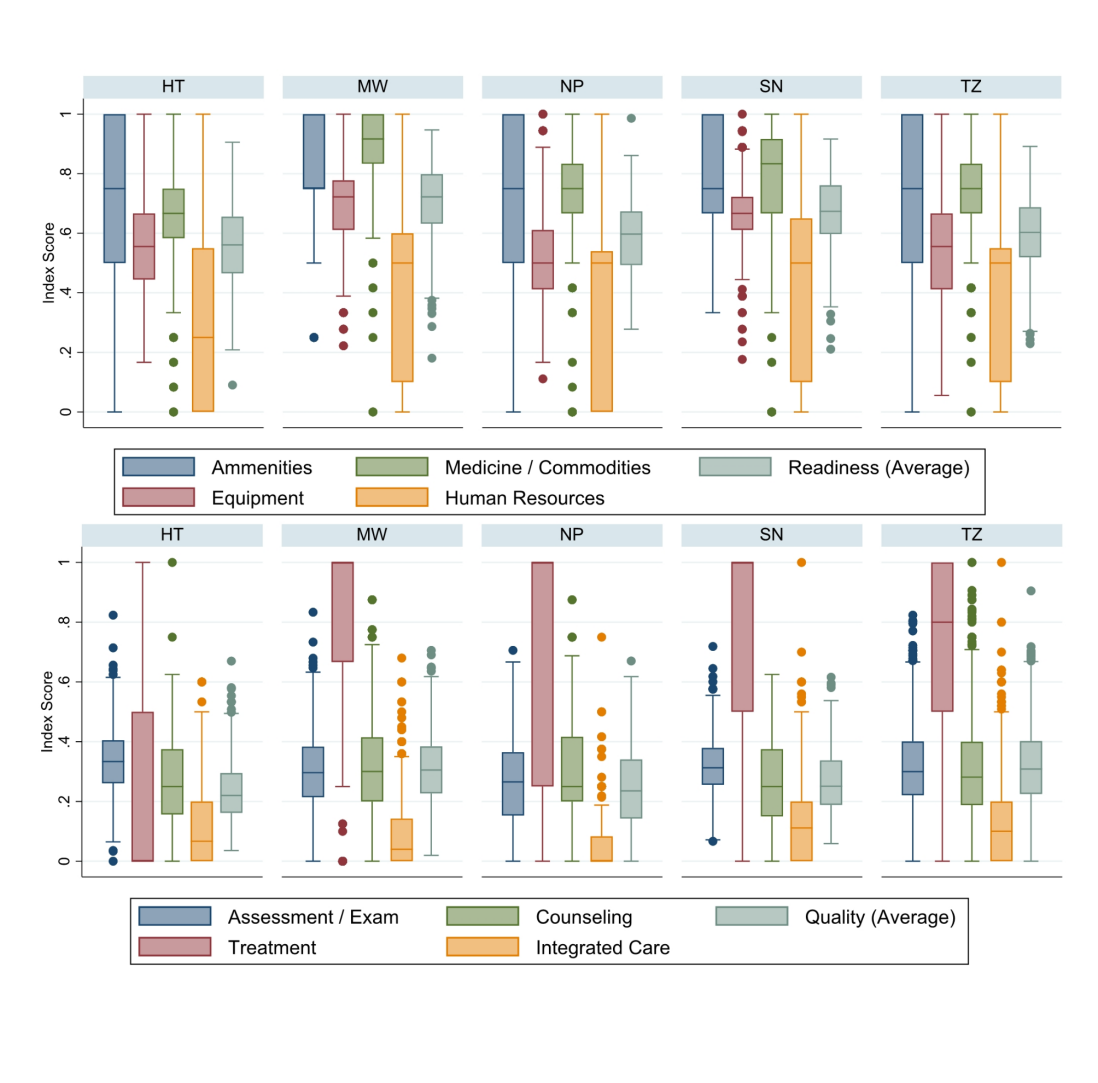
Distribution of facility-level domains of readiness and quality, by country

Figure 1 shows the distribution of facility-level readiness and quality scores by country. Facilities with at least one sick child observation that met the inclusion criteria and were included in the analysis had slightly better readiness scores than the full sample of facilities that treat sick children. Overall, median facility readiness ranged from 55% (Haiti) to 72% (Malawi). Median quality was substantially lower, ranging from 22% (Haiti) to 31% (Tanzania). Among those facilities included in the analysis, Fig. 2 shows the distribution of facility readiness and average facility quality by category of health facility. In general, both government and non-government referral facilities offered greater readiness than first-level facilities within a country. However, on average, first-level facilities provided a slightly better quality than referral facilities. Figure 3 shows the distribution of readiness and quality domains by country. Over half of all facilities in each country had amenities and commodities scores above 70% (i.e., 7 out of 10 items or better). The readiness domain with the worst performance was “human resources,” where three-quarters of facilities in each country had scores below 65%. Among the quality domains, most facilities performed poorly on assessment, counseling, and integrated care, with 75% of facilities on average performing fewer than half of the actions in each domain. Except for Haiti, most facilities had high ratings on the treatment domain. However, the variability in treatment was high, with an interquartile range exceeding 50% points in every country except for Malawi. For most children, only one or two treatment actions were recommended based on their diagnosis, or there was no assessable treatment action because the diagnosis did not align with a treatment action included in the observation protocol. This resulted in individual treatment scores that were heavily skewed to 0 or 1, or were absent entirely, contrary to the other domain values (Supplemental Fig. 1).

### Association between readiness and quality at the individual level

**Fig. 4:**
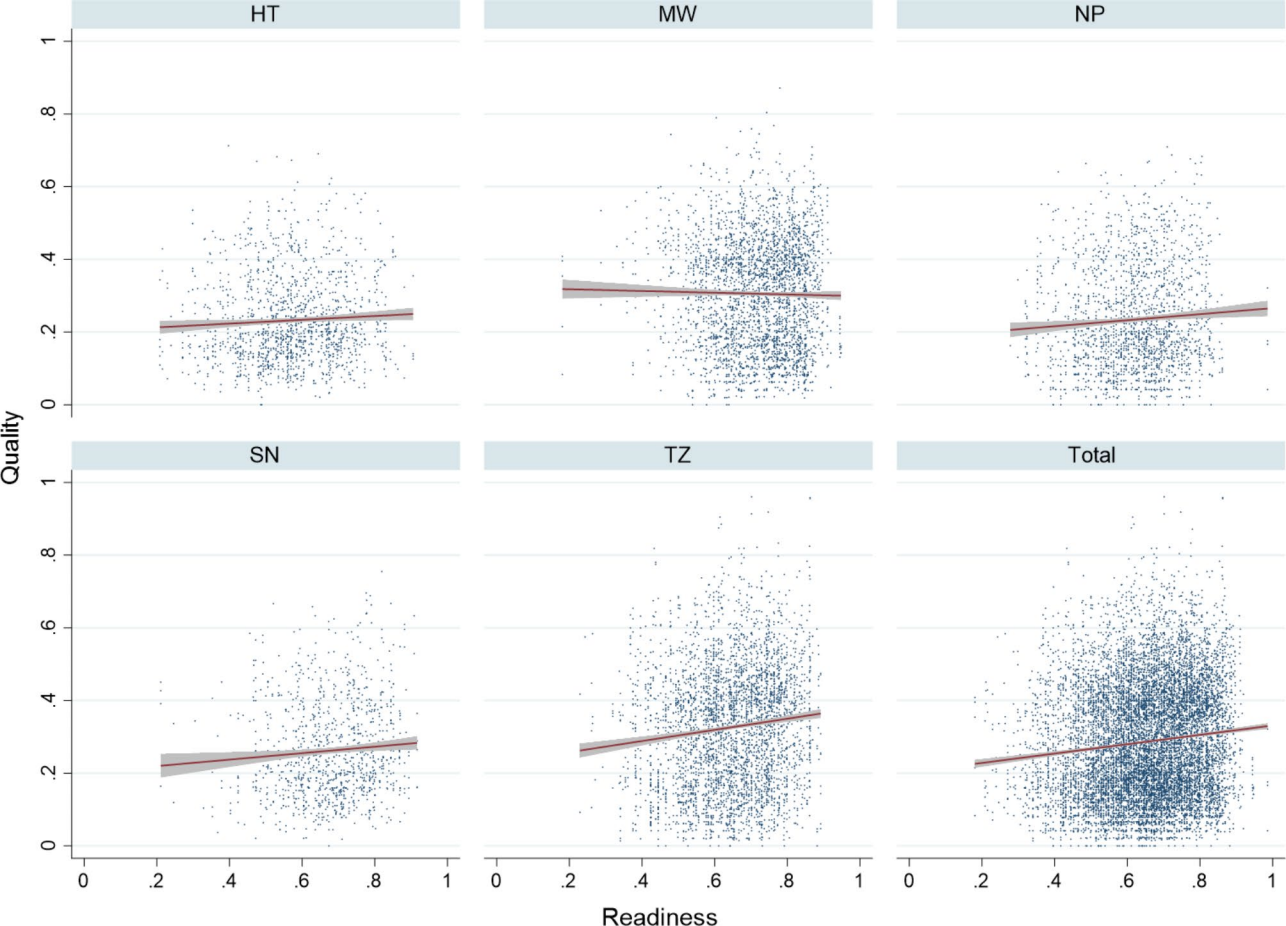
Unadjusted association between readiness and quality with linear fit for observations meeting inclusion criteria

We first assessed the overall association between facility readiness and quality for individual sick children with and without adjusting for other factors. Figure 4 shows the unadjusted correlation between facility readiness and the quality of individual care provided. Without adjusting for any other covariates, only Tanzania demonstrated a significant association between readiness and quality (0.146; 95% CI: 0.079–0.214), with each 10% point increase in readiness associated with a 1.46% point increase in quality (Table 5). After adjusting for facility type and managing authority, the magnitude of association between readiness and quality increased marginally in each of the five countries, and the association was significant in Haiti, Nepal, and Tanzania. For example, in Tanzania a 10% point increase in readiness was associated with a 1.62% point increase in quality after adjusting for facility type and managing authority.

**Table 5:** Association between readiness and quality, with and without adjusting for other covariates

Country	Unadjusted	Adjusting for facility type and managing authority	Adjusting for all covariates
	Coef (95% CI)	Coef (95% CI)	Coef (95% CI)
Haiti	0.038 (-0.025, 0.101)	0.088 (0.022, 0.155)	0.068 (0.005, 0.132)
Malawi	-0.038 (-0.107, 0.031)	0.034 (-0.042, 0.110)	0.056 (-0.014, 0.126)
Nepal	0.068 (-0.003, 0.138)	0.106 (0.035, 0.177)	0.083 (0.003, 0.164)
Senegal	0.087 (-0.003, 0.178)	0.094 (-0.003, 0.191)	0.123 (0.015, 0.23)
Tanzania	0.146 (0.079, 0.214)	0.162 (0.085, 0.238)	0.112 (0.043, 0.181)

Table 6 shows the unadjusted bivariate associations and adjusted association between quality and characteristics of the health facility (including readiness), health worker, and care-seeking episode. Covariates with categories that differed by country (i.e., region, facility type, and managing authority) are presented in Supplemental Table 1. After adjusting for characteristics of the facility, health worker, child, caretaker, and episode, every country except Malawi demonstrated a significant positive association between readiness and quality, ranging from 0.068 (95% CI: 0.005–0.132) in Haiti to 0.123 (95% CI: 0.015–0.23) in Senegal. In addition, a limited number of other factors were also associated with quality in multiple countries. In all five countries, increasing child age was associated with decreasing quality of care provided when adjusting for other factors. Additionally, having a diagnosis other than respiratory, digestive, or febrile illness was associated with poorer quality. In Malawi, Senegal, and Tanzania, increased cost of the visit was associated with better quality. These variables together, controlling for random effects, accounted for between 9% (Haiti) and 32% (Malawi) of the variation in quality scores (Supplemental Table 2).

**Table 6:**
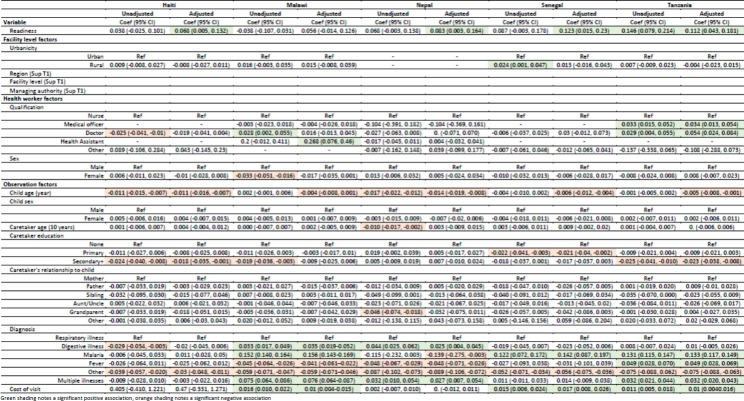
Association between quality and characteristics of the facility, health worker, patient, and illness episode

### Association by health facility category

We further examined the relationship between readiness and quality within health facility categories. Adjusting for finer delineations of type of provider and managing authority, we found a positive association between readiness and quality among first level government health facilities, the most common type of facility, in Nepal, Senegal, and Tanzania (Supplemental Table 3). This association ranged from 0.120 (95% CI: 0.014–0.227) in Senegal to 0.130 (95% CI: 0.033–0.226) in Tanzania. Additionally, in Tanzania, the magnitude of association between readiness and quality among non-government facilities was almost twice that observed in government facilities.

### Evaluating potential non-linear association

**Fig. 5:**
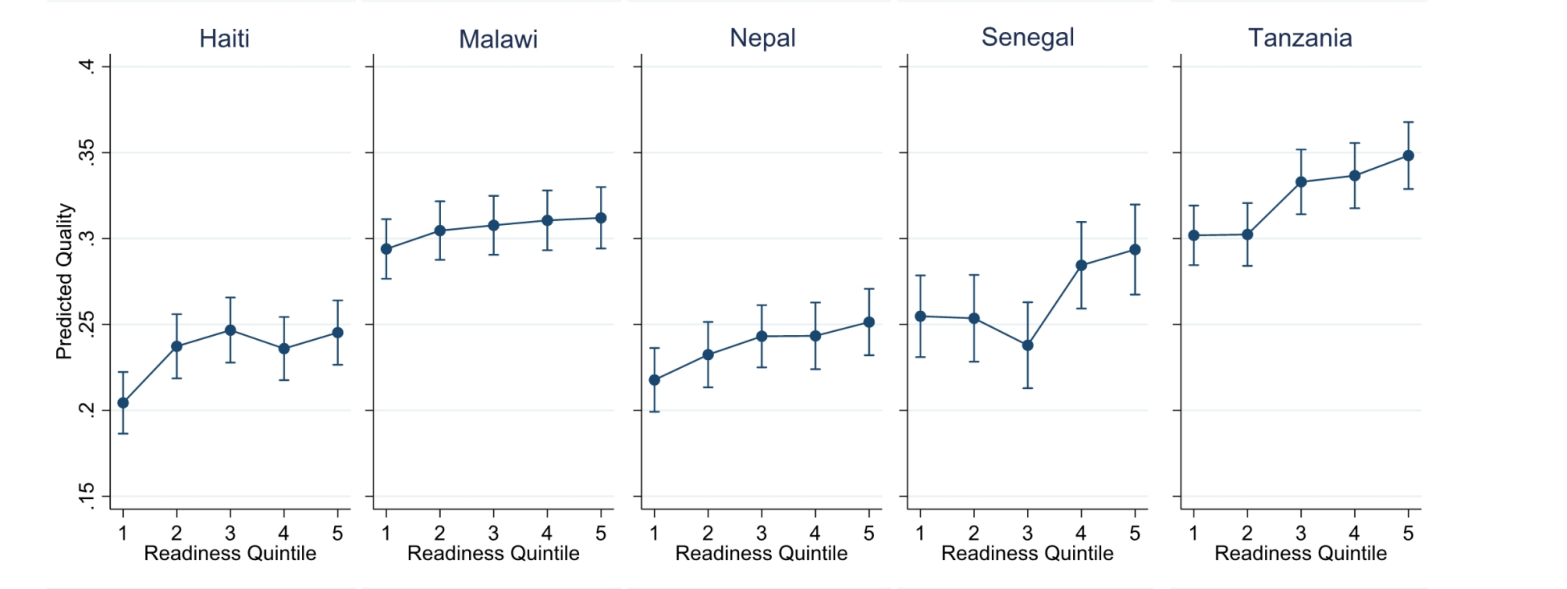
Predicted quality (with 95% CI) in each readiness quintile, adjusting for facility type and managing authority

We looked for a possible non-linear association between readiness and quality. Binning each facility into their relative readiness quintile by category of provider, we found limited evidence of a consistent non-linear association between readiness and quality (Fig. 5, Supplementary Table 4). In some countries, the association in the two upper or two lower quintiles did not increase linearly but instead plateaued. We also found no difference in the associations when considering absolute cut-offs for readiness, including readiness above and below 25%, 50%, and joints at each 20%, 40%, 60%, and 80% (data not shown).

### Association by index domains

Among domains of readiness (Table 7), we observed clear trends in the association with quality after adjusting for facility type and managing authority. In all countries except Malawi, the human resources domain was positively associated with the quality of care observed. The association ranged from 0.050 (95% CI: 0.013–0.087) in Senegal to 0.100 (95% CI: 0.067–0.132) in Tanzania, or about half of the magnitude to the total observed association between readiness and quality in those countries. Among the quality domains (Table 8), the quality of the assessment was significantly positively associated with readiness in four of the five countries, followed by quality of counseling and integrated care which both were significantly positively associated with readiness in three of the five countries. The treatment domain, however, was not significantly associated with readiness in any of the countries.

**Table 7:** Association between readiness domains and quality, adjusting for facility type and managing authority

Readiness Domain	Haiti	Malawi	Nepal	Senegal	Tanzania
	Coef (95% CI)	Coef (95% CI)	Coef (95% CI)	Coef (95% CI)	Coef (95% CI)
Amenities	-0.001 (-0.037, 0.035)	0.001 (-0.044, 0.045)	-0.007 (-0.055, 0.042)	0.016 (-0.054, 0.086)	-0.018 (-0.057, 0.021)
Equipment	0.062 (0.008, 0.117)	0.047 (-0.011, 0.106)	-0.007 (-0.058, 0.045)	-0.031 (-0.117, 0.054)	0.057 (0.008, 0.105)
Medicines / Commodities	-0.009 (-0.057, 0.039)	-0.010 (-0.081, 0.061)	0.008 (-0.045, 0.060)	0.011 (-0.046, 0.069)	0.043 (-0.015, 0.102)
Human Resources	0.056 (0.028, 0.083)	0.009 (-0.019, 0.038)	0.075 (0.045, 0.105)	0.050 (0.013, 0.087)	0.100 (0.067, 0.132)

**Table 8:** Association between readiness and quality domains, adjusting for facility type and managing authority

Quality Domain	Haiti	Malawi	Nepal	Senegal	Tanzania
	Coef (95% CI)	Coef (95% CI)	Coef (95% CI)	Coef (95% CI)	Coef (95% CI)
Examination / Assessment	0.120 (0.045, 0.195)	0.082 (0.001, 0.163)	0.129 (0.049, 0.208)	0.082 (-0.011, 0.174)	0.223 (0.144, 0.302)
Treatment	-0.012 (-0.293, 0.270)	0.081 (-0.110, 0.272)	0.002 (-0.335, 0.340)	-0.047 (-0.528, 0.435)	-0.103 (-0.297, 0.091)
Counselling	0.048 (-0.045, 0.141)	0.068 (-0.037, 0.174)	0.134 (0.031, 0.236)	0.167 (0.038, 0.297)	0.237 (0.131, 0.343)
Integrated Care	0.126 (0.034, 0.219)	0.007 (-0.071, 0.086)	0.076 (0.022, 0.131)	0.132 (-0.001, 0.265)	0.145 (0.061, 0.229)

### Association between readiness and quality (dropping treatment domain)

Without the treatment domain, we observed a significant positive association between readiness and quality in Nepal, Senegal, and Tanzania without adjusting for other factors (Supplemental Table 5). After adjusting for provider category alone, and fully adjusting for all covariates, we found a significant positive association between readiness and quality in all countries except for Malawi. We also observed a small increase (on average + 0.02) in the magnitude of the association when compared to the association using the quality score inclusive of the treatment domain.

Similar effects were observed when looking at the association by category of provider, with slight increases in the magnitude of association over those observed with the treatment-inclusive quality scores (Supplemental Table 6). Additional significant positive associations between readiness and quality, excluding the treatment domain, were observed among first-level government facilities and first-level non-government facilities in Malawi and Haiti, respectively.

Beyond the association between quality and facility readiness, we observed that most associations between characteristics of the facility, health worker, and quality remained stable when removing the treatment domain (Sup Tables 7 & 8). However, the association between the child’s diagnosis (reported by the provider) and quality was reduced when compared to the initial analysis. The overall amount of variation in quality explained by the characteristics was also substantially reduced (Supplemental Table 9).

Exclusion of the treatment domain did not alter the lack of evidence for a non-linear association between readiness and quality (Supplemental Table 10). It also did not change how domains of readiness were associated with quality, with human resources maintaining the same significant positive association with quality (Supplemental Table 11).

### Association between readiness and quality at the facility level

The results of the facility-level analysis did not vary greatly from the primary individual-level analysis (Supplemental Table 12). After adjusting for facility type and managing authority, all countries other than Malawi showed a significant positive association between readiness and quality. The magnitude of the association was slightly larger than that observed at the individual level in Senegal and Tanzania, and slightly lower in Haiti and Nepal. Further adjusting for region and urbanicity (the only facility-level covariates) reduced the magnitude of association, so that only Tanzania maintained a significant association between readiness and quality.

Similar results were seen when assessing association by facility category, with Senegal and Tanzania showing a greater magnitude of association among government first level providers compared to the individual assessment and weaker associations observed in Nepal (Supplemental Table 13).

The association at the domain level was not notably different from the individual level association, with the readiness human resources domain exhibiting the only significant association with quality (Supplemental Table 14), and the quality treatment domain lacking an association with readiness in all countries (Supplemental Table 15).

## Discussion

Taken as a whole, our three analyses suggest that facility readiness plays a role in the quality of case management provided to sick children but cannot serve as a proxy for clinical quality as currently measured. In four of the five countries included in the analysis, we observed a significant positive association between readiness and quality with a magnitude of approximately 0.1 after adjusting for facility type and managing authority. This suggests that within a provider category, a facility with a readiness score of 100% would have a quality score approximately 10% points higher than a facility with a readiness score of 0%. The observed association was most evident among first-level government facilities, although this may be due to sample size limitations in other provider categories. There was little evidence to suggest a non-linear relationship or a threshold effect altering the relationship between readiness and quality. Beyond readiness, younger child age (within the span of 2–59 m), higher cost of care, and having a respiratory, digestive, or febrile illness diagnosis were most often associated with a higher quality of care, in line with previously observed associations in other LMIC contexts [9–16].

The “human resources” readiness domain was consistently associated with quality, while the quality domain of “treatment” was the least influenced by readiness. Exclusion of the treatment domain from the quality score slightly increased the magnitude of association between readiness and quality, but did not alter the overall conclusions about the role of readiness in quality. After removing the treatment domain, we observed a reduction in the association between child’s diagnosis and quality, as well as the proportion of the overall variation in quality scores explained by the adjusted model. This was expected as the indicators contributing to the treatment domain were conditioned on diagnosis, and so the overall quality score was to some degree influenced by whether or not the child had a diagnosis that could be linked to an observed treatment for inclusion in the score. As noted in the methods section and below, the lack of influence of the “treatment” domain is likely an artifact of its poor definition based on the limited data and potentially biased data available. Repeating the analyses at a facility, rather than individual observation level, also did not change the overall conclusions.

Our results are similar to those found by Leslie and colleagues who examined the association between infrastructure and quality of child health care, among other health services, in eight primarily sub-Saharan African countries including four of the five countries included in our analysis [17]. The analysis, which looked exclusively at a facility-level association and did not adjust for facility, provider, or client characteristics, found a small positive linear correlation between infrastructure and process quality. Leslie’s analysis used indices derived from SPA indicators linked to WHO guidelines, and individual items differed somewhat from those derived from our expert survey. Their infrastructure score included a larger number of general amenities and fewer commodities, while the quality measure included fewer items related to integrated care and counseling, and no measure of treatment quality. However, the average readiness and quality scores, and trend in increasing quality with increased readiness, were similar to what we observed in our analysis. An analysis by Sheffel and colleagues in five countries found a threshold effect between readiness and provision of antenatal care suggesting that increasing readiness is associated with better quality below 50% readiness and at higher levels of readiness the relationship plateaus [19].

Our analysis used a robust measure of facility structural quality derived from SPA data. Using the results of the expert survey, we limited the number of structural quality items to 35, reflecting health facility amenities, equipment, commodities, and human resources deemed “essential” for the appropriate treatment of sick children. While the information collected by the SPA around facility readiness is extensive, some crucial items for sick child care are not included in the standard assessment in its current form, such as availability of mid-upper arm circumference tape and readiness-to-use therapeutic foods [21]. The amount of information collected on health worker training is also limited, and there is no assessment of their skills or knowledge. Given the relative importance of the human resources domain in explaining the quality observed in this analysis, more emphasis should be given to assessing health worker capacity to correctly manage sick children.

Many factors beyond structural capacity go into appropriate clinical care. Our analysis benefitted from an individual-level analysis, allowing for the isolation of the effect of facility readiness from other factors influencing the quality of care children received. In our analysis, much of the variation in the quality to sick children could not be explained by differences in facility readiness as currently measured. Our analysis suggests multiple factors, including child age, diagnosis, and cost of care, also affect the quality of care children receive. However, even accounting for a wide variety of health facility, health worker, child, caretaker, and illness episode characteristics only explained approximately 10% of the variation in quality of care. This suggests much of the variability in quality of care cannot be accounted for with the existing easy-to-assess measures and/or quality of care measurement issues complicate the identification of determinants. Identifying factors impacting these actions will also inform strategies to improve healthcare quality and ultimately save lives. Effective coverage cascades also utilize these constructs with the aim to understand the proportion of a population in need of an intervention that receives it with sufficient quality to achieve the intended health benefit. The standardized cascades proposed by the WHO Effective Coverage Think Tank Group, building off the Countdown to 2030, include both readiness (input-adjusted) and quality (quality-adjusted) coverage estimates as steps in the cascade [22, 23]. Within the context of operationalizing these cascades for application to sick child care in LMICs, it is essential to consider both how to define these constructs and understand how they relate to each other.

Our analysis and associated conclusions are limited by the data available for measuring provision of services. For defining observation-derived quality, we used the expert survey to prioritize essential actions. However, the potential indicators were limited by the mechanism of data collection and level of detail recorded. We included four domains of quality tied to IMCI guidelines for care of sick children including appropriate assessment (i.e., physical exam and history taking), treatment, counseling on illness management, and integrated care (e.g., delivery of preventative services and nutrition counseling) [20]. Notably absent are data on appropriate illness classification. The SPA observation protocol does not include any form of clinical reassessment [21], so it is not possible to ascertain if a health worker appropriately classified the child’s illness. Without this information, the subsequent step of appropriate treatment is fully conditional on the diagnosis given by the provider, which may be incorrect. The detail around the diagnosis is also limited, failing to note severity, and often grouping diagnoses in a manner that does not allow for determination of appropriate treatment. Further, the information on treatment administered is also limited with insufficient detail around the treatments (e.g., name of medicine) and dosage prescribed [21]. The lack of complete and verifiable data on whether a diagnosis was correct, and the subsequent treatment action was appropriate, potentially explains the lack of association between readiness and performance on the “treatment” domain in the quality index.

While administration of correct treatment is theoretically the most crucial aspect of appropriate care, at least in terms of immediate illness outcomes, the limited data available in the most common health facility surveys render the indicators uninformative. The revised SPA questionnaire (Q8), finalized in 2022, includes better information on facility case load, staff training, and select client-provider observation components, specifically documentation of reported fever, client’s current temperature, malaria RDT results, presence of palmar pallor and/or hemoglobin test results, and anthropometry [24]. However, the revised questionnaire still lacks documentation of other reported symptoms and the results of all other exam components preventing the data user from assessing the appropriateness of illness classification. Notably, despite the emphasis on improved quality of care assessment in the SPA redesign [25], clinical reassessment is still absent.

Beyond the survey indicator limitations, limited sample sizes may also have reduced our power to detect associations between readiness and quality, particularly within subgroups. Tanzania had the largest sample of both facilities and sick children allowing associations between readiness and quality stratifying by facility category to be measured with sufficient precision. This was not feasible in countries with smaller samples. We also did not account for multiple comparisons so a proportion of the statistically significant associations observed in the adjusted models may be Type I errors. However, the primary objective of the analysis was to characterize the association between readiness and quality and the interpretation of the role of characteristics of the health facility, health worker, child, caretaker, and illness episode were limited to cross-cutting or high level associations.

Our analysis only included data from five countries and a single time point, limiting the generalizability of the findings. We also limited our analysis to children aged 2 to 59 months that completed their visit and were either sent home or referred outside the facility. Insufficient data were available to accurately gauge the appropriate provision of services to children under 2 months, although these children are the most likely to suffer negative outcomes. Children admitted, sent for testing, or referred within the facility were lost to follow-up and excluded from the analysis due to incomplete observation data. However, these children may differ from those included in the sample based on underlying disease severity or complexity and ultimately might differ in the quality of care received. Finally, our readiness and quality indices were constructed around the IMCI guidelines which were developed to facilitate appropriate care in resource limited settings with less trained health workers [20]. The extent to which these guidelines are appropriate for higher-level facilities is debatable; however, the basic assessment, treatment, and counseling actions included in our index would be appropriate in most health care settings.

## Conclusions

These findings suggest facility readiness plays a role in clinical quality; however, the structural capacity to provide services is only one element influencing whether a health worker performs the appropriate actions to assess, classify, treat, counsel, and manage sick children. Investments beyond ensuring facilities are appropriately supplied are required to bridge the gap in high-quality health services in LMICs. As currently assessed, facility readiness cannot be used a proxy for quality. Data for measuring health facility readiness and quality of care are both limited, presenting challenges for gauging health system performance, assessing potential impediments to high-quality services, evaluating the impact of interventions, and monitoring changes in quality of care over time. We need better empiric data to assess the quality of care being delivered in these settings. Then additional work can be done to better understand what factors are driving quality of care and monitor quality improvement, with the ultimate goal of strengthening the management of sick children in these settings.

## Supplementary Information

Supplementary Material 1.

## Data Availability

The SPA datasets analyzed during the current study are available in the DHS repository, [https://dhsprogram.com/data/available-datasets.cfm].
