# Supplementary material for: Association between sick child facility readiness and quality of care at the individual and facility level in five low- and middle-income countries
Source: BMC Health Serv Res. 2024 Nov 14;24:1400. doi: 10.1186/s12913-024-11772-9 (PMC11562504; doi:10.1186/s12913-024-11772-9)
Supplement: Supplementary file 1 — Supplementary Material 1. [file 12913_2024_11772_MOESM1_ESM.docx]

Supplemental Figure 1. Distribution of individual observation provision of care domain scores

N/A

Supplemental Table 1. Association between provision of care and characteristics of the facility, health worker, patient, and illness episode – region, facility type, and managing authority

|  | **Haiti** | |  | **Malawi** | |  | **Nepal** | |
| --- | --- | --- | --- | --- | --- | --- | --- | --- |
|  | **Unadjusted** | **Adjusted** |  | **Unadjusted** | **Adjusted** |  | **Unadjusted** | **Adjusted** |
| **Region** | Coef (95% CI) | Coef (95% CI) | **Region** | Coef (95% CI) | Coef (95% CI) | **Region** | Coef (95% CI) | Coef (95% CI) |
| West | Ref | Ref | Chitipa | Ref | Ref | Eastern Mountain | Ref | Ref |
| Southeast | 0.04 (0.005-0.074) | 0.023 (-0.014-0.06) | Karonga | -0.016 (-0.093-0.061) | -0.028 (-0.096-0.04) | Central Mountain | -0.102 (-0.157–0.046) | -0.077 (-0.142–0.013) |
| North | -0.004 (-0.032-0.024) | -0.009 (-0.037-0.02) | Nkhata Bay | 0.056 (-0.02-0.131) | 0.009 (-0.058-0.076) | Western Mountain | -0.029 (-0.08-0.022) | -0.055 (-0.113-0.002) |
| Northeast | -0.024 (-0.067-0.019) | -0.033 (-0.077-0.01) | Rumphi | 0.045 (-0.036-0.125) | 0.021 (-0.051-0.092) | Eastern Hill | -0.082 (-0.131–0.033) | -0.044 (-0.102-0.013) |
| Artibonite | -0.017 (-0.041-0.008) | -0.033 (-0.059–0.006) | Mzimba | 0.041 (-0.027-0.11) | 0.03 (-0.031-0.091) | Central Hill | -0.097 (-0.141–0.052) | -0.077 (-0.13–0.025) |
| Central | 0.023 (-0.014-0.059) | 0.015 (-0.023-0.053) | Likoma | -0.088 (-0.246-0.07) | -0.109 (-0.251-0.033) | Western Hill | -0.084 (-0.13–0.038) | -0.061 (-0.115–0.008) |
| South | 0.018 (-0.012-0.048) | 0.001 (-0.029-0.032) | Kasungu | 0.038 (-0.034-0.109) | 0.017 (-0.046-0.08) | Mid-Western Hill | -0.075 (-0.123–0.027) | -0.071 (-0.125–0.017) |
| Grand-anse | 0.057 (0.023-0.092) | 0.044 (0.009-0.08) | Nkhotakota | 0.085 (0.01-0.16) | 0.035 (-0.031-0.102) | Far-Western Hill | -0.024 (-0.075-0.027) | -0.022 (-0.08-0.035) |
| Northwest | -0.027 (-0.057-0.003) | -0.043 (-0.076–0.011) | Ntchisi | 0.039 (-0.048-0.125) | 0.005 (-0.072-0.082) | Eastern Terai | -0.091 (-0.136–0.045) | -0.093 (-0.145–0.042) |
| Nippes | 0.023 (-0.019-0.065) | 0.01 (-0.033-0.053) | Dowa | 0.027 (-0.047-0.101) | 0.029 (-0.037-0.094) | Central Terai | -0.147 (-0.191–0.102) | -0.133 (-0.184–0.082) |
|  |  |  | Salima | 0.073 (-0.006-0.152) | 0.047 (-0.023-0.117) | Western Terai | -0.122 (-0.176–0.069) | -0.125 (-0.186–0.065) |
|  |  |  | Lilongwe | 0.057 (-0.009-0.124) | 0.027 (-0.033-0.087) | Mid-Western Terai | -0.062 (-0.113–0.011) | -0.078 (-0.135–0.021) |
|  |  |  | Mchinji | 0.093 (0.017-0.168) | 0.081 (0.012-0.149) | Far-Western Terai | -0.057 (-0.113-0.) | -0.066 (-0.127–0.004) |
|  |  |  | Dedza | 0.003 (-0.069-0.074) | -0.012 (-0.076-0.051) |  |  |  |
|  |  |  | Ntcheu | 0.041 (-0.033-0.115) | 0.021 (-0.044-0.087) |  |  |  |
|  |  |  | Mangochi | 0.039 (-0.03-0.109) | 0. (-0.062-0.061) |  |  |  |
|  |  |  | Machinga | 0.082 (0.003-0.162) | 0.049 (-0.023-0.12) |  |  |  |
|  |  |  | Zomba | 0.018 (-0.054-0.09) | 0.007 (-0.057-0.071) |  |  |  |
|  |  |  | Chiradzulu | 0.051 (-0.036-0.138) | -0.001 (-0.079-0.077) |  |  |  |
|  |  |  | Blantyre | 0.009 (-0.061-0.079) | 0. (-0.063-0.063) |  |  |  |
|  |  |  | Mwanza | 0.048 (-0.058-0.154) | 0.018 (-0.077-0.114) |  |  |  |
|  |  |  | Thyolo | -0.016 (-0.091-0.058) | -0.018 (-0.084-0.049) |  |  |  |
|  |  |  | Mulanje | -0.004 (-0.078-0.07) | -0.01 (-0.076-0.056) |  |  |  |
|  |  |  | Phalombe | 0.056 (-0.03-0.143) | 0.05 (-0.028-0.129) |  |  |  |
|  |  |  | Chikwawa | -0.05 (-0.129-0.028) | -0.06 (-0.131-0.01) |  |  |  |
|  |  |  | Nsanje | -0.006 (-0.09-0.079) | -0.015 (-0.091-0.062) |  |  |  |
|  |  |  | Balaka | 0.019 (-0.063-0.101) | -0.001 (-0.073-0.072) |  |  |  |
|  |  |  | Neno | 0.017 (-0.07-0.104) | -0.012 (-0.088-0.065) |  |  |  |
|  |  |  |  |  |  |  |  |  |
| **Facility type** |  |  | **Facility type** |  |  | **Facility type** |  |  |
| University hospital | Ref | Ref | Central hospital | Ref | Ref | Central gvt. Hospital | Ref | Ref |
| Regional hospital | 0.002 (-0.089-0.092) | -0.01 (-0.088-0.068) | District hospital | -0.013 (-0.115-0.089) | -0.039 (-0.129-0.052) | Regional gvt. Hospital | 0.009 (-0.132-0.15) | -0.125 (-0.307-0.057) |
| Community referral hospital | -0.012 (-0.081-0.057) | -0.007 (-0.064-0.051) | Rural / community hospital | 0.035 (-0.066-0.136) | -0.031 (-0.122-0.06) | Sub-regional gvt. Hospital | 0.085 (-0.036-0.206) | 0.064 (-0.11-0.238) |
| Hospital | -0.006 (-0.075-0.064) | 0.003 (-0.056-0.063) | Other hospital | 0.068 (-0.035-0.171) | 0.018 (-0.074-0.109) | Zonal govt. Hospital | 0.066 (-0.03-0.162) | 0.048 (-0.097-0.193) |
| Health center with lit | 0.012 (-0.053-0.077) | 0.018 (-0.038-0.074) | Health center | 0.049 (-0.047-0.146) | -0.004 (-0.091-0.083) | District govt. Hospital | 0.054 (-0.027-0.135) | 0.011 (-0.122-0.143) |
| Health center w/o lit | 0.016 (-0.047-0.079) | 0.019 (-0.035-0.074) | Dispensary | 0.078 (-0.025-0.181) | 0.02 (-0.072-0.113) | Other hospital | 0.082 (0.001-0.163) | -0.05 (-0.209-0.108) |
| Dispensary | 0.043 (-0.02-0.105) | 0.046 (-0.012-0.104) | Clinic | 0.066 (-0.032-0.164) | 0.019 (-0.073-0.111) | Primary health care center | 0.073 (-0.006-0.153) | 0.051 (-0.082-0.184) |
|  |  |  | Health post | 0.098 (-0.056-0.252) | -0.032 (-0.173-0.109) | Health post | 0.099 (0.02-0.179) | 0.067 (-0.066-0.2) |
|  |  |  |  |  |  | Sub-health post | 0.085 (0.002-0.167) | 0.077 (-0.059-0.213) |
|  |  |  |  |  |  | Urban health centre | 0.042 (-0.052-0.136) | 0.042 (-0.1-0.184) |
|  |  |  |  |  |  | Central level gvt. Hospital | 0.087 (-0.021-0.194) | 0.06 (-0.096-0.215) |
|  |  |  |  |  |  | District level gvt. Hospital | 0.054 (-0.036-0.145) | 0.036 (-0.109-0.182) |
|  |  |  |  |  |  |  |  |  |
| **Managing authority** |  |  | **Managing authority** |  |  | **Managing authority** |  |  |
| Government | Ref | Ref | Government | Ref | Ref | Government | Ref | Ref |
| NGO / private not for profit | 0.003 (-0.02-0.025) | 0.007 (-0.016-0.03) | Christian Health Association of Malawi (CHAM) | 0.036 (0.015-0.057) | 0.021 (0.-0.043) | NGO / private not for profit | -0.001 (-0.069-0.067) | 0.093 (-0.031-0.216) |
| Private for profit | 0.005 (-0.017-0.028) | 0.011 (-0.012-0.034) | Private for profit | 0.046 (0.022-0.07) | 0.004 (-0.036-0.045) | Private for profit | 0.011 (-0.019-0.041) | 0.113 (0.007-0.22) |
| Mission / faith-based | 0.006 (-0.015-0.027) | 0.001 (-0.02-0.021) | Mission / faith-based (not CHAM) | 0.103 (0.027-0.179) | 0.043 (-0.032-0.118) | Mission / faith-based | -0.079 (-0.183-0.025) | - |
|  |  |  | NGO | -0.015 (-0.057-0.027) | -0.02 (-0.069-0.029) |  |  |  |
|  |  |  | Company | -0.024 (-0.063-0.015) | -0.019 (-0.064-0.026) |  |  |  |

Green shading notes a significant positive association, orange shading notes a significant negative association.

Supplemental Table 1. Association between provision of care and characteristics of the facility, health worker, patient, and illness episode – region, facility type, and managing authority, continued

|  | **Senegal** | |  | **Tanzania** | |
| --- | --- | --- | --- | --- | --- |
|  | **Unadjusted** | **Adjusted** |  | **Unadjusted** | **Adjusted** |
| **Region** | Coef (95% CI) | Coef (95% CI) | **Region** | Coef (95% CI) | Coef (95% CI) |
| Dakar | Ref | Ref | Dodoma | Ref | Ref |
| Diourbel | 0.017 (-0.033-0.068) | -0.005 (-0.062-0.051) | Arusha | 0.104 (0.048-0.161) | 0.093 (0.041-0.146) |
| Fatick | -0.023 (-0.073-0.027) | -0.054 (-0.111-0.002) | Kilimanjaro | 0.037 (-0.019-0.093) | 0.037 (-0.016-0.089) |
| Kaffrine | 0.072 (0.023-0.122) | 0.051 (-0.005-0.108) | Tanga | 0.008 (-0.051-0.067) | -0.015 (-0.07-0.04) |
| Kaokack | 0.005 (-0.047-0.057) | -0.024 (-0.081-0.034) | Morogoro | 0.048 (-0.017-0.112) | 0.032 (-0.029-0.092) |
| Kedougou | 0.009 (-0.051-0.069) | -0.045 (-0.115-0.025) | Pwani | -0.082 (-0.158–0.007) | -0.086 (-0.159–0.014) |
| Kolda | -0.056 (-0.104–0.008) | -0.077 (-0.13–0.024) | Dar Es Salaam | -0.015 (-0.071-0.04) | -0.031 (-0.085-0.023) |
| Louga | 0.028 (-0.026-0.082) | 0.021 (-0.037-0.08) | Lindi | 0.007 (-0.051-0.065) | -0.003 (-0.057-0.05) |
| Matam | 0.058 (0.011-0.106) | 0.033 (-0.021-0.087) | Mtwara | 0.074 (0.016-0.132) | 0.037 (-0.017-0.091) |
| Saint louis | 0.032 (-0.017-0.081) | 0.012 (-0.041-0.065) | Ruvuma | 0.061 (0.005-0.117) | 0.012 (-0.039-0.064) |
| Sediou | -0.025 (-0.074-0.025) | -0.046 (-0.107-0.014) | Iringa | 0.084 (0.024-0.144) | 0.064 (0.009-0.119) |
| Tambacounda | -0.038 (-0.092-0.017) | -0.051 (-0.112-0.01) | Mbeya | 0.007 (-0.051-0.066) | 0.005 (-0.049-0.059) |
| Thies | 0. (-0.038-0.037) | -0.026 (-0.069-0.018) | Singida | 0.12 (0.063-0.177) | 0.119 (0.067-0.171) |
| Ziguinchor | 0.009 (-0.037-0.056) | -0.002 (-0.054-0.05) | Tabora | 0.046 (-0.011-0.103) | 0.015 (-0.038-0.068) |
|  |  |  | Rukwa | -0.022 (-0.087-0.043) | -0.034 (-0.094-0.027) |
|  |  |  | Kigoma | 0.083 (0.023-0.142) | 0.046 (-0.008-0.101) |
|  |  |  | Shinyanga | 0.194 (0.133-0.255) | 0.161 (0.104-0.218) |
|  |  |  | Kagera | 0.098 (0.041-0.155) | 0.063 (0.011-0.116) |
|  |  |  | Mwanza | 0.097 (0.041-0.153) | 0.076 (0.025-0.128) |
|  |  |  | Mara | -0.004 (-0.067-0.059) | -0.012 (-0.07-0.046) |
|  |  |  | Manyara | 0.061 (0.001-0.121) | 0.066 (0.011-0.122) |
|  |  |  | Njombe | 0.035 (-0.026-0.097) | 0.039 (-0.017-0.095) |
|  |  |  | Katavi | 0.002 (-0.059-0.062) | -0.014 (-0.07-0.042) |
|  |  |  | Simiyu | 0.048 (-0.013-0.109) | 0.03 (-0.027-0.087) |
|  |  |  | Geita | 0.125 (0.058-0.192) | 0.087 (0.025-0.149) |
|  |  |  | Kaskazini Unguja | 0.067 (-0.001-0.134) | 0.087 (0.024-0.149) |
|  |  |  | Kusini Unguja | 0.04 (-0.027-0.107) | 0.074 (0.012-0.137) |
|  |  |  | Mjini Magharib | -0.001 (-0.068-0.067) | 0.014 (-0.052-0.079) |
|  |  |  | Kaskazini Pemba | 0.001 (-0.064-0.066) | 0.035 (-0.024-0.094) |
|  |  |  | Kusini Pemba | -0.051 (-0.115-0.014) | -0.007 (-0.066-0.053) |
|  |  |  |  |  |  |
| **Facility type** |  |  | **Facility type** |  |  |
| Hospital | Ref | Ref | National referral hospital | Ref | Ref |
| Health center | 0.004 (-0.038-0.047) | 0.019 (-0.035-0.074) | Regional hospital | 0.05 (-0.052-0.151) | -0.024 (-0.108-0.06) |
| Clinic | 0.021 (-0.017-0.058) | 0.042 (-0.013-0.096) | District hospital | 0.041 (-0.051-0.132) | -0.021 (-0.098-0.057) |
|  |  |  | District-designated hospital | 0.009 (-0.093-0.111) | -0.073 (-0.161-0.014) |
|  |  |  | Other hospital (private) | 0.078 (-0.012-0.168) | -0.004 (-0.081-0.074) |
|  |  |  | Health centre | 0.057 (-0.031-0.145) | -0.003 (-0.079-0.073) |
|  |  |  | Clinic | 0.043 (-0.059-0.145) | 0.033 (-0.057-0.123) |
|  |  |  | Dispensary | 0.053 (-0.035-0.14) | 0.013 (-0.064-0.09) |
|  |  |  |  |  |  |
| **Managing authority** |  |  | **Managing authority** |  |  |
| Government | Ref | Ref | Government/public | Ref | Ref |
| NGO / private not for profit | 0.037 (-0.143-0.218) | 0.03 (-0.141-0.201) | Private-for-profit | 0.014 (-0.014-0.041) | 0.007 (-0.025-0.04) |
| Private for profit | -0.029 (-0.073-0.015) | 0. (-0.049-0.049) | Mission / faith-based | 0.039 (0.017-0.061) | 0.017 (-0.008-0.042) |
| Mission / faith-based | -0.003 (-0.056-0.049) | -0.001 (-0.053-0.05) | Parastatal | 0.018 (-0.055-0.091) | 0.03 (-0.038-0.097) |

Green shading notes a significant positive association, orange shading notes a significant negative association.

Supplemental Table 2. Proportion of variance in provision of care explained by each set of covariates

| **Country** | **Unadjusted** | **Adjusting for Facility type** | **Adjusting for all covariates** |
| --- | --- | --- | --- |
| Haiti | 0.04% | 1.68% | 9.06% |
| Malawi | 0.04% | 0.96% | 32.38% |
| Nepal | 0.10% | 1.11% | 21.48% |
| Senegal | 0.27% | 0.45% | 14.47% |
| Tanzania | 0.42% | 0.83% | 20.72% |

Supplemental Table 3. Association between readiness and provision of care by facility type and managing authority

| **Country** | **Govt Referral** | **Govt First Level** | **Non-Govt Referral** | **Non-Govt First Level** |
| --- | --- | --- | --- | --- |
|  | Coef (95% CI) | Coef (95% CI) | Coef (95% CI) | Coef (95% CI) |
| Haiti | 0.191 (-0.042-0.423) | 0.088 (-0.023-0.199) | 0.145 (-0.052-0.342) | 0.053 (-0.042-0.149) |
| Malawi | 0.282 (-0.021-0.585) | 0.103 (-0.006-0.212) | 0.131 (-0.261-0.523) | -0.094 (-0.210-0.022) |
| Nepal | 0.144 (0.002-0.285) | 0.125 (0.029-0.222) | - | 0.002 (-0.122-0.126) |
| Senegal | 0.228 (-0.041-0.497) | 0.120 (0.014-0.227) | -0.110 (-0.528-0.309) | -0.112 (-0.420-0.196) |
| Tanzania | 0.145 (-0.096-0.386) | 0.130 (0.033-0.226) | 0.272 (0.003-0.542) | 0.294 (0.142-0.446) |

Supplemental Table 4. Association between provision of care and readiness by quintile, adjusting for facility type and managing authority

| **Readiness Quintile** | **Haiti** | **Malawi** | **Nepal** | **Senegal** | **Tanzania** |
| --- | --- | --- | --- | --- | --- |
|  | Coef (95% CI) | Coef (95% CI) | Coef (95% CI) | Coef (95% CI) | Coef (95% CI) |
| 1 (Lowest 20%) | -0.043 (-0.068- -0.018) | -0.014 (-0.038-0.011) | -0.025 (-0.051-0.000) | 0.017 (-0.018-0.051) | -0.031 (-0.057- -0.006) |
| 2 | -0.012 (-0.037-0.013) | -0.003 (-0.027-0.021) | -0.011 (-0.037-0.016) | 0.016 (-0.020-0.051) | -0.031 (-0.057- -0.004) |
| 3 (Reference) | - | - | - | - | - |
| 4 | -0.009 (-0.034-0.016) | 0.003 (-0.021-0.027) | 0.000 (-0.026-0.027) | 0.047 (0.011-0.082) | 0.004 (-0.023-0.030) |
| 5 (Highest 20%) | -0.002 (-0.027-0.023) | 0.004 (-0.020-0.029) | 0.008 (-0.018-0.035) | 0.056 (0.020-0.092) | 0.015 (-0.012-0.042) |

Supplemental Table 5. Association between readiness and provision of care, excluding treatment domain

| **Country** | **Unadjusted** | **Adjusting for facility type and managing authority** | **Adjusting for all covariates** |
| --- | --- | --- | --- |
|  | Coef (95% CI) | Coef (95% CI) | Coef (95% CI) |
| Haiti | 0.051 (-0.007-0.108) | 0.097 (0.035-0.159) | 0.086 (0.022-0.149) |
| Malawi | -0.018 (-0.079-0.044) | 0.047 (-0.020-0.114) | 0.043 (-0.023-0.110) |
| Nepal | 0.067 (0.007-0.126) | 0.106 (0.043-0.169) | 0.102 (0.035-0.168) |
| Senegal | 0.146 (0.056-0.236) | 0.126 (0.037-0.215) | 0.112 (0.014-0.211) |
| Tanzania | 0.168 (0.103-0.233) | 0.198 (0.124-0.272) | 0.141 (0.074-0.208) |

Supplemental Table 6. Association between readiness and provision of care by provider category, excluding treatment domain

| **Country** | **Govt Referral** | **Govt First Level** | **Non-Govt Referral** | **Non-Govt First Level** |
| --- | --- | --- | --- | --- |
|  | Coef (95% CI) | Coef (95% CI) | Coef (95% CI) | Coef (95% CI) |
| Haiti | 0.187 (-0.024-0.399) | 0.067 (-0.035-0.169) | 0.104 (-0.107-0.316) | 0.099 (0.010-0.188) |
| Malawi | 0.166 (-0.103-0.435) | 0.128 (0.031-0.226) | 0.145 (-0.193-0.483) | -0.064 (-0.165-0.038) |
| Nepal | 0.151 (0.036-0.266) | 0.132 (0.053-0.211) | - | -0.032 (-0.172-0.107) |
| Senegal | 0.066 (-0.187-0.319) | 0.178 (0.081-0.275) | -0.268 (-0.776-0.240) | -0.058 (-0.402-0.286) |
| Tanzania | 0.124 (-0.103-0.351) | 0.188 (0.098-0.279) | 0.205 (-0.061-0.471) | 0.329 (0.179-0.478) |

Supplemental Table 7. Association between provision of care and characteristics of the facility, health worker, patient, and illness episode – excluding treatment domain

|  | **Haiti** | | **Malawi** | | **Nepal** | | **Senegal** | | **Tanzania** | |
| --- | --- | --- | --- | --- | --- | --- | --- | --- | --- | --- |
|  | **Unadjusted** | **Adjusted** | **Unadjusted** | **Adjusted** | **Unadjusted** | **Adjusted** | **Unadjusted** | **Adjusted** | **Unadjusted** | **Adjusted** |
| **Variable** | Coef (95% CI) | Coef (95% CI) | Coef (95% CI) | Coef (95% CI) | Coef (95% CI) | Coef (95% CI) | Coef (95% CI) | Coef (95% CI) | Coef (95% CI) | Coef (95% CI) |
| Readiness | 0.05 (-0.011-0.111) | 0.086 (0.022-0.149) | -0.018 (-0.079-0.044) | 0.043 (-0.023-0.11) | 0.067 (0.007-0.126) | 0.102 (0.035-0.168) | 0.146 (0.056-0.236) | 0.112 (0.014-0.211) | 0.168 (0.103-0.233) | 0.141 (0.074-0.208) |
| **Facility level factors** |  |  |  |  |  |  |  |  |  |  |
| Urbanicity |  |  |  |  |  |  |  |  |  |  |
| Urban | Ref | Ref | Ref | Ref | Ref | Ref | Ref | Ref | Ref | Ref |
| Rural | 0.01 (-0.006-0.025) | -0.004 (-0.023-0.015) | 0.002 (-0.015-0.019) | 0.016 (-0.006-0.038) | - | - | 0.02 (-0.002-0.041) | 0.018 (-0.01-0.045) | 0.001 (-0.015-0.017) | -0.005 (-0.024-0.014) |
| Region |  |  |  |  |  |  |  |  |  |  |
| Facility level |  |  |  |  |  |  |  |  |  |  |
| Managing authority |  |  |  |  |  |  |  |  |  |  |
| **Health worker factors** |  |  |  |  |  |  |  |  |  |  |
| Qualification |  |  |  |  |  |  |  |  |  |  |
| Nurse | Ref | Ref | Ref | Ref | Ref | Ref | Ref | Ref | Ref | Ref |
| Medical officer | - | - | -0.018 (-0.036-0.) | -0.016 (-0.037-0.005) | -0.007 (-0.237-0.222) | 0.01 (-0.208-0.227) | - | - | 0.027 (0.009-0.045) | 0.028 (0.007-0.048) |
| Doctor | -0.025 (-0.04–0.01) | -0.025 (-0.047–0.004) | 0.027 (0.004-0.05) | 0.011 (-0.016-0.038) | 0.011 (-0.02-0.042) | 0.027 (-0.03-0.085) | 0.001 (-0.028-0.031) | 0.032 (-0.01-0.073) | 0.035 (0.01-0.059) | 0.046 (0.017-0.075) |
| Health Assistant | - | - | - | - | -0.006 (-0.029-0.017) | 0.01 (-0.02-0.041) | - | - | - | - |
| Other | 0.02 (-0.167-0.208) | -0.039 (-0.219-0.141) | 0.097 (-0.095-0.288) | 0.129 (-0.058-0.315) | -0.024 (-0.149-0.1) | 0.027 (-0.089-0.142) | -0.028 (-0.079-0.023) | -0.029 (-0.079-0.022) | -0.07 (-0.26-0.121) | -0.08 (-0.254-0.094) |
| Sex |  |  |  |  |  |  |  |  |  |  |
| Male | Ref | Ref | Ref | Ref | Ref | Ref | Ref | Ref | Ref | Ref |
| Female | 0.008 (-0.008-0.024) | -0.008 (-0.026-0.009) | -0.018 (-0.034–0.003) | -0.012 (-0.029-0.005) | 0.012 (-0.004-0.028) | 0.007 (-0.018-0.032) | -0.000 (-0.022-0.021) | -0.001 (-0.023-0.021) | -0.006 (-0.021-0.009) | 0.007 (-0.008-0.022) |
| **Observation factors** |  |  |  |  |  |  |  |  |  |  |
| Child age (year) | -0.011 (-0.014–0.007) | -0.01 (-0.014–0.006) | -0.004 (-0.006–0.001) | -0.004 (-0.006–0.001) | -0.013 (-0.016–0.009) | -0.009 (-0.014–0.005) | -0.008 (-0.013–0.003) | -0.008 (-0.013–0.002) | -0.007 (-0.009–0.004) | -0.007 (-0.009–0.004) |
| Chidl sex |  |  |  |  |  |  |  |  |  |  |
| Male | Ref | Ref | Ref | Ref | Ref | Ref | Ref | Ref | Ref | Ref |
| Female | 0.006 (-0.003-0.016) | 0.004 (-0.005-0.014) | 0.008 (0.002-0.013) | 0.008 (0.002-0.013) | -0.004 (-0.012-0.005) | -0.007 (-0.017-0.003) | -0.004 (-0.016-0.008) | -0.006 (-0.019-0.007) | 0.001 (-0.005-0.008) | 0.001 (-0.005-0.008) |
| Caretaker age (10 years) | -0.003 (-0.009-0.002) | -0.001 (-0.008-0.006) | -0.002 (-0.006-0.002) | 0. (-0.004-0.005) | -0.006 (-0.012–0.001) | 0. (-0.009-0.01) | 0.000 (-0.006-0.007) | 0.008 (-0.001-0.018) | -0.004 (-0.008-0.000) | -0.003 (-0.008-0.002) |
| Caretaker education |  |  |  |  |  |  |  |  |  |  |
| None | Ref | Ref | Ref | Ref | Ref | Ref | Ref | Ref | Ref | Ref |
| Primary | -0.007 (-0.021-0.007) | -0.008 (-0.022-0.006) | -0.002 (-0.011-0.007) | -0.015 (-0.03-0.001) | 0.014 (-0.002-0.029) | 0.003 (-0.015-0.02) | -0.021 (-0.036–0.005) | -0.019 (-0.035–0.002) | -0.008 (-0.017-0.001) | -0.011 (-0.021–0.002) |
| Secondary+ | -0.018 (-0.032–0.004) | -0.015 (-0.029–0.001) | -0.003 (-0.014-0.007) | -0.001 (-0.011-0.009) | 0.008 (-0.003-0.019) | 0.005 (-0.009-0.018) | -0.017 (-0.032–0.001) | -0.012 (-0.029-0.005) | -0.012 (-0.024–0.000) | -0.016 (-0.028–0.004) |
| Caretaker’s relationship to child |  |  |  |  |  |  |  |  |  |  |
| Mother | Ref | Ref | Ref | Ref | Ref | Ref | Ref | Ref | Ref | Ref |
| Father | -0.004 (-0.026-0.018) | 0. (-0.022-0.023) | -0.015 (-0.030-0.000) | -0.004 (-0.015-0.007) | -0.013 (-0.028-0.003) | 0.004 (-0.015-0.023) | -0.026 (-0.050–0.002) | -0.024 (-0.051-0.003) | -0.008 (-0.023-0.006) | 0.003 (-0.012-0.018) |
| Sibling | -0.028 (-0.081-0.025) | -0.017 (-0.069-0.034) | 0.001 (-0.009-0.012) | -0.001 (-0.012-0.01) | -0.039 (-0.076–0.001) | -0.021 (-0.062-0.019) | -0.027 (-0.069-0.015) | -0.008 (-0.052-0.037) | -0.019 (-0.045-0.007) | -0.014 (-0.039-0.012) |
| Aunt/Uncle | -0.003 (-0.026-0.020) | 0. (-0.022-0.023) | -0.029 (-0.057–0.001) | -0.025 (-0.053-0.003) | -0.001 (-0.037-0.035) | -0.003 (-0.039-0.033) | -0.035 (-0.062–0.008) | -0.03 (-0.058–0.002) | -0.003 (-0.037-0.031) | 0.011 (-0.023-0.045) |
| Grandparent | -0.013 (-0.036-0.009) | -0.011 (-0.038-0.017) | -0.018 (-0.038-0.003) | -0.016 (-0.041-0.009) | -0.031 (-0.052–0.010) | -0.017 (-0.051-0.016) | -0.023 (-0.048-0.003) | -0.029 (-0.067-0.01) | -0.010 (-0.031-0.011) | 0.005 (-0.019-0.029) |
| Other | 0.013 (-0.017-0.044) | 0.021 (-0.009-0.052) | 0.004 (-0.016-0.024) | -0.002 (-0.022-0.019) | -0.031 (-0.124-0.061) | -0.012 (-0.102-0.078) | 0.045 (-0.084-0.174) | 0.1 (-0.028-0.229) | -0.016 (-0.055-0.023) | -0.009 (-0.047-0.029) |
| Diagnosis |  |  |  |  |  |  |  |  |  |  |
| Respiratory illness | Ref | Ref | Ref | Ref | Ref | Ref | Ref | Ref | Ref | Ref |
| Digestive illness | 0.008 (-0.014-0.030) | 0.012 (-0.009-0.034) | 0.008 (-0.003-0.020) | 0.007 (-0.004-0.019) | 0.007 (-0.007-0.021) | 0.003 (-0.013-0.02) | 0.009 (-0.014-0.031) | 0.002 (-0.023-0.027) | 0.025 (0.012-0.037) | 0.027 (0.015-0.039) |
| Malaria | -0.015 (-0.048-0.018) | -0.004 (-0.038-0.029) | 0.015 (0.006-0.024) | 0.019 (0.01-0.029) | -0.032 (-0.121-0.057) | -0.024 (-0.13-0.082) | -0.024 (-0.068-0.019) | -0.018 (-0.066-0.03) | 0.023 (0.011-0.036) | 0.028 (0.015-0.04) |
| Fever | -0.013 (-0.045-0.018) | -0.013 (-0.044-0.018) | -0.001 (-0.015-0.012) | -0.002 (-0.015-0.012) | -0.014 (-0.029-0.000) | -0.009 (-0.027-0.008) | 0.009 (-0.047-0.066) | -0.002 (-0.063-0.058) | -0.006 (-0.023-0.010) | -0.005 (-0.021-0.012) |
| Other | -0.030 (-0.045–0.014) | -0.023 (-0.039–0.008) | -0.034 (-0.043–0.025) | -0.033 (-0.042–0.024) | -0.062 (-0.074–0.051) | -0.055 (-0.069–0.042) | -0.018 (-0.034–0.002) | -0.021 (-0.038–0.004) | -0.028 (-0.038–0.017) | -0.027 (-0.038–0.017) |
| Multiple illnesses | 0.007 (-0.010-0.023) | 0.01 (-0.006-0.027) | 0.011 (0.003-0.019) | 0.01 (0.002-0.018) | 0.016 (-0.001-0.033) | 0.024 (0.002-0.045) | 0.015 (-0.004-0.034) | 0.017 (-0.003-0.038) | 0.017 (0.008-0.026) | 0.017 (0.008-0.026) |
| Cost of visit | 0.351 (-0.377-1.079) | 0.414 (-0.307-1.134) | 0.012 (0.008-0.017) | 0.007 (0.003-0.012) | 0.002 (-0.005-0.009) | -0.001 (-0.009-0.008) | 0.008 (0.000-0.015) | 0.012 (0.004-0.02) | 0.008 (0.002-0.013) | 0.006 (0.001-0.012) |

Green shading notes a significant positive association, orange shading notes a significant negative association.

Supplemental Table 8. Association between provision of care and characteristics of the facility, health worker, patient, and illness episode – region, facility type, and managing authority

|  | **Haiti** | |  | **Malawi** | |  | **Nepal** | |
| --- | --- | --- | --- | --- | --- | --- | --- | --- |
|  | **Unadjusted** | **Adjusted** |  | **Unadjusted** | **Adjusted** |  | **Unadjusted** | **Adjusted** |
| **Region** | Coef (95% CI) | Coef (95% CI) | **Region** | Coef (95% CI) | Coef (95% CI) | **Region** | Coef (95% CI) | Coef (95% CI) |
| West | Ref | Ref | Chitipa | Ref | Ref | Eastern Mountain | Ref | Ref |
| Southeast | 0.038 (0.003-0.073) | 0.018 (-0.019-0.054) | Karonga | -0.006 (-0.073-0.062) | -0.011 (-0.077-0.054) | Central Mountain | -0.076 (-0.124--0.027) | -0.067 (-0.121--0.013) |
| North | -0.014 (-0.044-0.015) | -0.023 (-0.053-0.006) | Nkhata Bay | 0.039 (-0.028-0.105) | 0.028 (-0.036-0.093) | Western Mountain | -0.028 (-0.073-0.017) | -0.043 (-0.092-0.005) |
| Northeast | -0.024 (-0.069-0.02) | -0.038 (-0.082-0.006) | Rumphi | 0.036 (-0.035-0.107) | 0.039 (-0.029-0.108) | Eastern Hill | -0.06 (-0.103--0.016) | -0.043 (-0.091-0.006) |
| Artibonite | -0.014 (-0.04-0.011) | -0.038 (-0.065--0.011) | Mzimba | 0.074 (0.014-0.134) | 0.063 (0.005-0.122) | Central Hill | -0.063 (-0.103--0.024) | -0.061 (-0.105--0.017) |
| Central | 0.027 (-0.01-0.063) | 0.009 (-0.028-0.047) | Likoma | -0.041 (-0.184-0.102) | -0.055 (-0.194-0.083) | Western Hill | -0.053 (-0.094--0.012) | -0.047 (-0.092--0.003) |
| South | 0.013 (-0.018-0.044) | -0.003 (-0.035-0.028) | Kasungu | 0.038 (-0.024-0.101) | 0.03 (-0.03-0.091) | Mid-Western Hill | -0.06 (-0.102--0.018) | -0.059 (-0.104--0.014) |
| Grand-anse | 0.053 (0.017-0.09) | 0.033 (-0.004-0.07) | Nkhotakota | 0.08 (0.014-0.146) | 0.066 (0.002-0.13) | Far-Western Hill | -0.026 (-0.071-0.02) | -0.04 (-0.088-0.008) |
| Northwest | -0.029 (-0.06-0.001) | -0.052 (-0.085--0.02) | Ntchisi | 0.035 (-0.042-0.112) | 0.029 (-0.046-0.103) | Eastern Terai | -0.063 (-0.103--0.023) | -0.069 (-0.113--0.026) |
| Nippes | 0.012 (-0.031-0.055) | -0.008 (-0.051-0.035) | Dowa | 0.058 (-0.007-0.123) | 0.053 (-0.01-0.116) | Central Terai | -0.122 (-0.161--0.082) | -0.117 (-0.16--0.075) |
|  |  |  | Salima | 0.064 (-0.005-0.133) | 0.062 (-0.005-0.129) | Western Terai | -0.104 (-0.15--0.057) | -0.119 (-0.169--0.068) |
|  |  |  | Lilongwe | 0.07 (0.011-0.128) | 0.048 (-0.01-0.105) | Mid-Western Terai | -0.048 (-0.093--0.003) | -0.072 (-0.12--0.024) |
|  |  |  | Mchinji | 0.107 (0.041-0.173) | 0.107 (0.041-0.173) | Far-Western Terai | -0.055 (-0.104--0.005) | -0.067 (-0.118--0.015) |
|  |  |  | Dedza | 0.029 (-0.034-0.092) | 0.016 (-0.045-0.077) |  |  |  |
|  |  |  | Ntcheu | 0.052 (-0.013-0.117) | 0.043 (-0.02-0.106) |  |  |  |
|  |  |  | Mangochi | 0.039 (-0.021-0.1) | 0.024 (-0.035-0.084) |  |  |  |
|  |  |  | Machinga | 0.075 (0.005-0.145) | 0.057 (-0.011-0.126) |  |  |  |
|  |  |  | Zomba | 0.032 (-0.031-0.096) | 0.02 (-0.042-0.081) |  |  |  |
|  |  |  | Chiradzulu | 0.046 (-0.031-0.122) | 0.028 (-0.046-0.103) |  |  |  |
|  |  |  | Blantyre | 0.046 (-0.014-0.107) | 0.035 (-0.026-0.095) |  |  |  |
|  |  |  | Mwanza | 0.048 (-0.044-0.14) | 0.042 (-0.05-0.134) |  |  |  |
|  |  |  | Thyolo | 0.032 (-0.033-0.097) | 0.028 (-0.036-0.092) |  |  |  |
|  |  |  | Mulanje | 0.028 (-0.037-0.093) | 0.021 (-0.043-0.085) |  |  |  |
|  |  |  | Phalombe | 0.087 (0.011-0.164) | 0.081 (0.005-0.156) |  |  |  |
|  |  |  | Chikwawa | -0.022 (-0.09-0.047) | -0.033 (-0.101-0.034) |  |  |  |
|  |  |  | Nsanje | 0.006 (-0.069-0.08) | -0.011 (-0.084-0.062) |  |  |  |
|  |  |  | Balaka | 0.037 (-0.035-0.109) | 0.029 (-0.04-0.099) |  |  |  |
|  |  |  | Neno | 0.019 (-0.057-0.095) | 0.018 (-0.056-0.091) |  |  |  |
|  |  |  |  |  |  |  |  |  |
| **Facility type** |  |  | **Facility type** |  |  | **Facility type** |  |  |
| University hospital | Ref | Ref | Central hospital | Ref | Ref | Central gvt. Hospital | Ref | Ref |
| Regional hospital | -0.012 (-0.09-0.065) | 0.005 (-0.081-0.092) | District hospital | -0.097 (-0.185--0.009) | -0.082 (-0.168-0.003) | Regional gvt. Hospital | -0.01 (-0.152-0.132) | -0.08 (-0.233-0.073) |
| Community referral hospital | 0. (-0.059-0.058) | 0.002 (-0.063-0.068) | Rural / community hospital | -0.038 (-0.124-0.049) | -0.061 (-0.147-0.024) | Sub-regional gvt. Hospital | 0.054 (-0.071-0.179) | 0.022 (-0.118-0.163) |
| Hospital | -0.002 (-0.061-0.057) | 0.011 (-0.055-0.078) | Other hospital | 0.007 (-0.081-0.095) | -0.01 (-0.096-0.077) | Zonal govt. Hospital | 0.06 (-0.04-0.161) | 0.064 (-0.056-0.183) |
| Health center with lit | 0.016 (-0.04-0.071) | 0.027 (-0.035-0.09) | Health center | -0.033 (-0.116-0.049) | -0.043 (-0.124-0.039) | District govt. Hospital | 0.037 (-0.052-0.126) | 0.013 (-0.094-0.119) |
| Health center w/o lit | 0.017 (-0.037-0.07) | 0.021 (-0.041-0.082) | Dispensary | -0.029 (-0.117-0.06) | -0.031 (-0.118-0.057) | Other hospital | 0.087 (-0.002-0.175) | -0.008 (-0.133-0.117) |
| Dispensary | 0.042 (-0.011-0.096) | 0.046 (-0.018-0.111) | Clinic | -0.007 (-0.091-0.076) | -0.024 (-0.111-0.063) | Primary health care center | 0.055 (-0.032-0.141) | 0.051 (-0.056-0.159) |
|  |  |  | Health post | -0.004 (-0.141-0.133) | -0.071 (-0.207-0.065) | Health post | 0.073 (-0.013-0.16) | 0.059 (-0.048-0.167) |
|  |  |  |  |  |  | Sub-health post | 0.054 (-0.034-0.143) | 0.065 (-0.045-0.175) |
|  |  |  |  |  |  | Urban health centre | 0.036 (-0.06-0.133) | 0.049 (-0.067-0.164) |
|  |  |  |  |  |  | Central level gvt. Hospital | 0.086 (-0.029-0.201) | 0.083 (-0.042-0.208) |
|  |  |  |  |  |  | District level gvt. Hospital | 0.025 (-0.071-0.12) | -0.001 (-0.119-0.118) |
|  |  |  |  |  |  |  |  |  |
| **Managing authority** |  |  | **Managing authority** |  |  | **Managing authority** |  |  |
| Government | Ref | Ref | Government | Ref | Ref | Government | Ref | Ref |
| NGO / private not for profit | 0.002 (-0.019-0.024) | 0.002 (-0.022-0.025) | Christian Health Association of Malawi (CHAM) | 0.036 (0.017-0.055) | 0.02 (0.-0.041) | NGO / private not for profit | 0.016 (-0.042-0.074) | 0.051 (-0.043-0.145) |
| Private for profit | 0.007 (-0.014-0.029) | 0.004 (-0.019-0.027) | Private for profit | 0.048 (0.027-0.069) | 0.018 (-0.02-0.057) | Private for profit | 0.035 (0.01-0.06) | 0.074 (-0.009-0.157) |
| Mission / faith-based | 0.001 (-0.02-0.021) | -0.006 (-0.027-0.015) | Mission / faith-based (not CHAM) | 0.083 (0.012-0.154) | 0.024 (-0.047-0.094) | Mission / faith-based | -0.024 (-0.111-0.064) | - |
|  |  |  | NGO | 0.011 (-0.025-0.048) | -0.023 (-0.069-0.023) |  |  |  |
|  |  |  | Company | 0.01 (-0.024-0.043) | 0.006 (-0.037-0.048) |  |  |  |

Green shading notes a significant positive association, orange shading notes a significant negative association.

Supplemental Table 8. Association between provision of care and characteristics of the facility, health worker, patient, and illness episode – region, facility type, and managing authority, continued

|  | **Senegal** | |  | **Tanzania** | |
| --- | --- | --- | --- | --- | --- |
|  | **Unadjusted** | **Adjusted** |  | **Unadjusted** | **Adjusted** |
| **Region** | Coef (95% CI) | Coef (95% CI) | **Region** | Coef (95% CI) | Coef (95% CI) |
| Dakar | Ref | Ref | Dodoma | Ref | Ref |
| Diourbel | -0.001 (-0.049-0.047) | -0.028 (-0.079-0.024) | Arusha | 0.15 (0.097-0.203) | 0.142 (0.092-0.193) |
| Fatick | -0.02 (-0.067-0.027) | -0.052 (-0.104-0.) | Kilimanjaro | 0.057 (0.005-0.109) | 0.047 (-0.003-0.097) |
| Kaffrine | 0.055 (0.008-0.101) | 0.023 (-0.03-0.075) | Tanga | 0.011 (-0.044-0.066) | -0.008 (-0.061-0.045) |
| Kaokack | -0.013 (-0.062-0.036) | -0.043 (-0.094-0.008) | Morogoro | 0.061 (0.001-0.121) | 0.048 (-0.01-0.107) |
| Kedougou | -0.032 (-0.088-0.024) | -0.058 (-0.124-0.007) | Pwani | -0.067 (-0.136-0.003) | -0.067 (-0.136-0.002) |
| Kolda | -0.052 (-0.098--0.006) | -0.067 (-0.117--0.018) | Dar Es Salaam | 0.026 (-0.026-0.078) | 0.005 (-0.046-0.057) |
| Louga | 0.048 (-0.002-0.099) | 0.038 (-0.016-0.093) | Lindi | -0.003 (-0.058-0.051) | -0.004 (-0.056-0.048) |
| Matam | 0.056 (0.01-0.101) | 0.026 (-0.024-0.076) | Mtwara | 0.045 (-0.01-0.099) | 0.037 (-0.016-0.089) |
| Saint louis | 0.041 (-0.006-0.087) | 0.014 (-0.035-0.064) | Ruvuma | 0.036 (-0.017-0.088) | 0.014 (-0.036-0.063) |
| Sediou | -0.025 (-0.072-0.022) | -0.055 (-0.111-0.001) | Iringa | 0.12 (0.064-0.175) | 0.097 (0.044-0.15) |
| Tambacounda | -0.05 (-0.101-0.002) | -0.055 (-0.113-0.002) | Mbeya | 0.036 (-0.018-0.091) | 0.032 (-0.02-0.085) |
| Thies | -0.009 (-0.044-0.027) | -0.034 (-0.074-0.005) | Singida | 0.141 (0.087-0.195) | 0.13 (0.079-0.181) |
| Ziguinchor | 0.013 (-0.031-0.057) | 0.004 (-0.044-0.052) | Tabora | 0.051 (-0.003-0.105) | 0.03 (-0.021-0.082) |
|  |  |  | Rukwa | 0.005 (-0.056-0.065) | -0.009 (-0.067-0.05) |
|  |  |  | Kigoma | 0.044 (-0.012-0.1) | 0.024 (-0.029-0.077) |
|  |  |  | Shinyanga | 0.242 (0.185-0.299) | 0.223 (0.168-0.278) |
|  |  |  | Kagera | 0.083 (0.03-0.137) | 0.07 (0.02-0.121) |
|  |  |  | Mwanza | 0.094 (0.042-0.147) | 0.084 (0.034-0.134) |
|  |  |  | Mara | -0.01 (-0.069-0.049) | -0.016 (-0.072-0.041) |
|  |  |  | Manyara | 0.083 (0.026-0.14) | 0.082 (0.028-0.135) |
|  |  |  | Njombe | 0.048 (-0.009-0.105) | 0.049 (-0.005-0.103) |
|  |  |  | Katavi | 0.003 (-0.054-0.06) | -0.007 (-0.061-0.048) |
|  |  |  | Simiyu | 0.058 (0.001-0.116) | 0.04 (-0.015-0.095) |
|  |  |  | Geita | 0.118 (0.055-0.181) | 0.094 (0.034-0.155) |
|  |  |  | Kaskazini Unguja | 0.116 (0.051-0.18) | 0.117 (0.056-0.179) |
|  |  |  | Kusini Unguja | 0.076 (0.013-0.139) | 0.087 (0.027-0.148) |
|  |  |  | Mjini Magharib | 0.028 (-0.035-0.09) | 0.036 (-0.027-0.099) |
|  |  |  | Kaskazini Pemba | 0.019 (-0.042-0.081) | 0.034 (-0.023-0.092) |
|  |  |  | Kusini Pemba | -0.028 (-0.089-0.034) | -0.001 (-0.059-0.057) |
|  |  |  |  |  |  |
| **Facility type** |  |  | **Facility type** |  |  |
| Hospital | Ref | Ref | National referral hospital | Ref | Ref |
| Health center | -0.012 (-0.053-0.028) | 0.007 (-0.043-0.057) | Regional hospital | 0.036 (-0.05-0.123) | -0.022 (-0.103-0.06) |
| Clinic | 0.008 (-0.028-0.044) | 0.029 (-0.021-0.079) | District hospital | 0.033 (-0.045-0.112) | 0.004 (-0.071-0.078) |
|  |  |  | District-designated hospital | 0.029 (-0.059-0.117) | -0.015 (-0.099-0.069) |
|  |  |  | Other hospital (private) | 0.07 (-0.008-0.147) | 0.027 (-0.049-0.102) |
|  |  |  | Health centre | 0.048 (-0.028-0.124) | 0.024 (-0.049-0.098) |
|  |  |  | Clinic | 0.046 (-0.043-0.135) | 0.07 (-0.017-0.157) |
|  |  |  | Dispensary | 0.045 (-0.031-0.12) | 0.045 (-0.029-0.119) |
|  |  |  |  |  |  |
| **Managing authority** |  |  | **Managing authority** |  |  |
| Government | Ref | Ref | Government/public | Ref | Ref |
| NGO / private not for profit | 0.056 (-0.093-0.206) | 0.056 (-0.087-0.199) | Private-for-profit | 0.016 (-0.011-0.044) | -0.011 (-0.042-0.02) |
| Private for profit | -0.024 (-0.064-0.017) | -0.014 (-0.058-0.031) | Mission / faith-based | 0.025 (0.002-0.048) | 0.001 (-0.023-0.025) |
| Mission / faith-based | -0.002 (-0.048-0.044) | -0.001 (-0.047-0.045) | Parastatal | 0.022 (-0.053-0.097) | 0.02 (-0.046-0.085) |

Green shading notes a significant positive association, orange shading notes a significant negative association.

Supplemental Table 9. Proportion of variance in provision of care explained by each set of covariates, excluding treatment domain

| **Country** | **Unadjusted** | **Adjusting for facility type and managing authority** | **Adjusting for all covariates** |
| --- | --- | --- | --- |
| Haiti | 0.05% | 1.26% | 10.28% |
| Malawi | 0.02% | 0.54% | 8.46% |
| Nepal | 0.21% | 1.73% | 11.55% |
| Senegal | 0.52% | 0.63% | 6.43% |
| Tanzania | 0.33% | 0.51% | 8.66% |

Supplemental Table 10. Association between provision of care and readiness by quintile, adjusting for facility type and managing authority, excluding treatment domain

| **Readiness Quintile** | **Haiti** | **Malawi** | **Nepal** | **Senegal** | **Tanzania** |
| --- | --- | --- | --- | --- | --- |
|  | Coef (95% CI) | Coef (95% CI) | Coef (95% CI) | Coef (95% CI) | Coef (95% CI) |
| 1 (Lowest 20%) | -0.039 (-0.063–0.015) | -0.013 (-0.035-0.008) | -0.021 (-0.043-0.001) | 0.001 (-0.031-0.033) | -0.034 (-0.057–0.010) |
| 2 | -0.016 (-0.040-0.008) | 0.003 (-0.019-0.024) | 0.002 (-0.020-0.024) | 0.018 (-0.015-0.050) | -0.03 (-0.054–0.006) |
| 3 (Reference) | - | - | - | - | - |
| 4 | -0.01 (-0.034-0.015) | 0.006 (-0.016-0.027) | 0 (-0.023-0.022) | 0.029 (-0.003-0.062) | 0.008 (-0.017-0.032) |
| 5 (Highest 20%) | 0.002 (-0.023-0.026) | 0.006 (-0.016-0.028) | 0.021 (-0.001-0.044) | 0.044 (0.011-0.077) | 0.022 (-0.002-0.047) |

Supplemental Table 11. Association between readiness domains and provision of care, adjusting for facility type and managing authority, excluding treatment domain

| **Readiness Domain** | **Haiti** | **Malawi** | **Nepal** | **Senegal** | **Tanzania** |
| --- | --- | --- | --- | --- | --- |
|  | Coef (95% CI) | Coef (95% CI) | Coef (95% CI) | Coef (95% CI) | Coef (95% CI) |
| Amenities | 0.006 (-0.030-0.042) | -0.01 (-0.049-0.030) | 0.002 (-0.040-0.044) | 0.01 (-0.057-0.077) | -0.022 (-0.060-0.016) |
| Equipment | 0.055 (-0.001-0.111) | 0.039 (-0.014-0.091) | 0.014 (-0.030-0.057) | 0.018 (-0.064-0.099) | 0.095 (0.049-0.142) |
| Medicines / Commodities | -0.009 (-0.053-0.035) | 0.023 (-0.039-0.085) | 0.009 (-0.035-0.054) | 0.028 (-0.028-0.084) | 0.036 (-0.021-0.094) |
| Human Resources | 0.058 (0.032-0.084) | 0.018 (-0.008-0.044) | 0.065 (0.039-0.091) | 0.068 (0.032-0.104) | 0.117 (0.084-0.150) |

Supplemental Table 12. Association between readiness and provision of care at facility level

| **Country** | **Unadjusted** | **Adjusting for facility type and managing authority** | **Adjusting for all covariates** |
| --- | --- | --- | --- |
|  | Coef (95% CI) | Coef (95% CI) | Coef (95% CI) |
| Haiti | 0.031 (-0.033-0.096) | 0.085 (0.014-0.155) | 0.065 (-0.004-0.133) |
| Malawi | -0.045 (-0.116-0.025) | 0.025 (-0.056-0.107) | 0.029 (-0.052-0.110) |
| Nepal | 0.052 (-0.024-0.127) | 0.086 (0.004-0.168) | 0.060 (-0.022-0.141) |
| Senegal | 0.128 (0.018-0.239) | 0.114 (0.001-0.228) | 0.047 (-0.068-0.162) |
| Tanzania | 0.223 (0.123-0.322) | 0.238 (0.127-0.349) | 0.193 (0.085-0.301) |

Supplemental Table 13. Association between readiness and provision of care at facility level by provider category

| **Country** | **Govt Referral** | **Govt First Level** | **Non-Govt Referral** | **Non-Govt First Level** |
| --- | --- | --- | --- | --- |
|  | Coef (95% CI) | Coef (95% CI) | Coef (95% CI) | Coef (95% CI) |
| Haiti | 0.166 (-0.074-0.405) | 0.060 (-0.060-0.179) | 0.184 (-0.026-0.394) | 0.040 (-0.057-0.136) |
| Malawi | 0.298 (-0.061-0.657) | 0.100 (-0.012-0.212) | 0.154 (-0.286-0.594) | -0.118 (-0.226–0.009) |
| Nepal | 0.128 (-0.036-0.292) | 0.075 (-0.019-0.170) | - | -0.023 (-0.202-0.156) |
| Senegal | 0.197 (-0.298-0.693) | 0.141 (0.030-0.252) | -0.027 (-0.499-0.445) | 0.097 (-0.433-0.627) |
| Tanzania | 0.147 (-0.099-0.393) | 0.195 (0.068-0.321) | 0.343 (-0.008-0.693) | 0.339 (0.105-0.573) |

Supplemental Table 14. Association between readiness domains and provision of care at facility level, adjusting for facility type and managing authority

| **Readiness Domain** | **Haiti** | **Malawi** | **Nepal** | **Senegal** | **Tanzania** |
| --- | --- | --- | --- | --- | --- |
|  | Coef (95% CI) | Coef (95% CI) | Coef (95% CI) | Coef (95% CI) | Coef (95% CI) |
| Amenities | 0.000 (-0.039-0.039) | -0.003 (-0.050-0.044) | -0.007 (-0.059-0.046) | 0.026 (-0.056-0.107) | 0.011 (-0.041-0.063) |
| Equipment | 0.043 (-0.015-0.102) | 0.038 (-0.026-0.101) | -0.017 (-0.074-0.040) | 0.014 (-0.083-0.112) | 0.025 (-0.053-0.103) |
| Medicines / Commodities | -0.007 (-0.058-0.044) | -0.024 (-0.097-0.049) | 0.006 (-0.053-0.065) | 0.021 (-0.051-0.094) | 0.067 (-0.033-0.166) |
| Human Resources | 0.055 (0.022-0.087) | 0.011 (-0.020-0.041) | 0.065 (0.032-0.099) | 0.049 (0.007-0.091) | 0.149 (0.103-0.195) |

Supplemental Table 15. Association between readiness and provision of care domains at facility level, adjusting for facility type and managing authority

| **Provision of care Domain** | **Haiti** | **Malawi** | **Nepal** | **Senegal** | **Tanzania** |
| --- | --- | --- | --- | --- | --- |
|  | Coef (95% CI) | Coef (95% CI) | Coef (95% CI) | Coef (95% CI) | Coef (95% CI) |
| Examination / Assessment | 0.106 (0.028-0.185) | 0.077 (-0.010-0.164) | 0.111 (0.022-0.200) | 0.106 (-0.001-0.213) | 0.249 (0.136-0.363) |
| Treatment | -0.004 (-0.306-0.298) | 0.107 (-0.120-0.333) | 0.011 (-0.364-0.385) | -0.163 (-0.685-0.360) | 0.073 (-0.257-0.403) |
| Counselling | 0.046 (-0.058-0.150) | 0.067 (-0.046-0.179) | 0.121 (0.012-0.231) | 0.198 (0.052-0.344) | 0.379 (0.215-0.542) |
| Integrated Care | 0.127 (0.027-0.226) | -0.001 (-0.084-0.083) | 0.052 (-0.012-0.116) | 0.199 (0.055-0.344) | 0.186 (0.053-0.318) |

**Supplementary Box 1. Readiness Indicators and Ranking**

| **Proportion rank essential** | **Domain** | **Item** | | **Outcome** |
| --- | --- | --- | --- | --- |
| 0.8 | Amenities | Improved water source | | Included |
| 0.725 | Amenities | Emergency transportation | | Included |
| 0.675 | Amenities | Sanitation facilities | | Included |
| 0.625 | Amenities | Power | | Included |
| 0.375 | Amenities | Communication equipment | |  |
| 0.325 | Amenities | Room with auditory and visual privacy | |  |
| 0.15 | Amenities | Computer with email/internet access | |  |
| 0.85 | Equipment | Soap and water | | Included |
| 0.8 | Equipment | Infant weighing scale | | Included |
| 0.775 | Equipment | Thermometer | | Included |
| 0.775 | Equipment | Child weighing scale | | Included |
| 0.725 | Equipment | Bag and mask | | Included |
| 0.7 | Equipment | Single use syringes | | Included |
| 0.675 | Equipment | Equipment to test for malaria | | Included |
| 0.675 | Equipment | Disinfectant | | Included |
| 0.625 | Equipment | Timer, watch, or device that can measure seconds | | Included |
| 0.625 | Equipment | Medical masks | | Included |
| 0.6 | Equipment | Oxygen equipment (concentrator OR cylinder OR distribution system) | | Included |
| 0.575 | Equipment | Stethoscope | | Included |
| 0.575 | Equipment | Latex gloves | | Included |
| 0.575 | Equipment | Appropriate storage of sharps waste (sharps box) | | Included |
| 0.55 | Equipment | Cannula for administering IV fluids | | Included |
| 0.55 | Equipment | Alcohol based hand rub | | Included |
| 0.55 | Equipment | MUAC tape | | Included |
| 0.55 | Equipment | Appropriate storage of infectious waste (pedal bin with lid and liner) | | Included |
| 0.5 | Equipment | Height or length board | | Included |
| 0.45 | Equipment | Safe final disposal of sharps (incineration) | |  |
| 0.425 | Equipment | Equipment to test blood oxygen saturation (pulse oximeter) | | Included for consistency |
| 0.425 | Equipment | Calibrated 1/2 or 1 | |  |
| 0.425 | Equipment | Safe final disposal of infectious waste (incineration) | |  |
| 0.375 | Equipment | Equipment to test bronchodilator response (nebulizer) | |  |
| 0.375 | Equipment | Examination table/bed | |  |
| 0.35 | Equipment | Nebulizer | |  |
| 0.325 | Equipment | Cup and spoon | |  |
| 0.325 | Equipment | Eye protection (goggles or face protection) | |  |
| 0.275 | Equipment | Spacer for inhaler | |  |
| 0.25 | Equipment | Gowns | |  |
| 0.2 | Equipment | Equipment to perform stool culture (solutions and microscope + electricity) | |  |
| 0.125 | Equipment | Equipment to examine chest infiltrates or pleural effusions (x-ray and supplies) | |  |
| 0.8 | Human Resources | At least one clinical staff member trained in sick child care (IMCI and/or diagnosis and/or treatment of malaria, ARI, diarrhea and/or micronutrient deficiencies) in the last two years | | Included |
| 0.6 | Human Resources | Any guidelines for diagnosis and management of childhood illnesses available at the facility | | Included |
| 0.45 | Human Resources | At least 75% of clinical staff offering child curative services at the facility have received supervision in the last six months | |  |
| 0.425 | Human Resources | At least one clinical staff member completed in-service IMCI training in the last two years | |  |
| 0.425 | Human Resources | IMCI guidelines available at the facility | |  |
| 0.3 | Human Resources | At least 75% of clinical staff offering child curative services at the facility have received supervision WITH OBSERVATION in the last six months | |  |
| 0.15 | Human Resources | At least 75% of clinical staff offering child curative services at the facility report availability of opportunities for promotion in their current job | |  |
| 0.9 | Medicines/Commodities | Oral rehydration therapy (ORS) | | Included |
| 0.85 | Medicines/Commodities | First-line antimalarial - in malaria endemic areas (oral - artemisinin combination therapy (ACT), chloroquine in Haiti) | | Included |
| 0.65 | Medicines/Commodities | Amoxicillin and/or cotrimoxazole (oral) | | Included |
| 0.65 | Medicines/Commodities | IV fluids (Ringers lactate, normal saline, and/or 5% dextrose) | | Included |
| 0.6 | Medicines/Commodities | Any injectable antibiotic (ampicillin, ceftriaxone, gentamycin, or penicillin) | | Included |
| 0.6 | Medicines/Commodities | Injectable antimalarial - in malaria endemic areas (Quinine, Artesunate) | | Included |
| 0.575 | Medicines/Commodities | Zinc (oral) |  | Included |
| 0.575 | Medicines/Commodities | Paracetamol (oral) | | Included |
| 0.55 | Medicines/Commodities | Vitamin A (oral) | | Included |
| 0.525 | Medicines/Commodities | Bronchodilator or salbutamol inhaler | | Included |
| 0.525 | Medicines/Commodities | De-worming drugs (oral - mebendazole, albendazole) | | Included |
| 0.475 | Medicines/Commodities | Gentamycin (injectable) | |  |
| 0.425 | Medicines/Commodities | Diazepam (injectable) | | Included for consistency |
| 0.4 | Medicines/Commodities | Ceftriaxone (injectable) | |  |
| 0.4 | Medicines/Commodities | Tetracycline eye ointment (TEO) | |  |
| 0.375 | Medicines/Commodities | Ampicillin (injectable) | |  |
| 0.35 | Medicines/Commodities | Iron tablets (oral) | |  |
| 0.3 | Medicines/Commodities | Rectal antimalarial - in malaria endemic areas (Artesunate suppository) | |  |
| 0.3 | Medicines/Commodities | Metronidazole (oral) | |  |
| 0.25 | Medicines/Commodities | Benzylpenicillin (injectable) | |  |
| 0.225 | Medicines/Commodities | Augmentin (oral) | |  |
| 0.2 | Medicines/Commodities | Azithromycin, erythromycin, or tetracycline (oral) | |  |
| 0.2 | Medicines/Commodities | Doxyclycline (oral) | |  |
| 0.2 | Medicines/Commodities | Nystatin (oral) | |  |
| 0.175 | Medicines/Commodities | Ciprofloxin (oral) | |  |
| 0.1 | Medicines/Commodities | Cefixime (oral) | |  |

**Supplementary Box 2. Provision of Care Indicators and Ranking**

| **Proportion rank essential** | **Domain** | **Item** | **Outcome** |
| --- | --- | --- | --- |
| 0.95 | Assessment | Assessed history of cough or difficult breathing | Included |
| 0.95 | Assessment | Assessed history of child unable to drink or breastfeed during illness | Included |
| 0.9 | Assessment | Assessed history of fever | Included |
| 0.85 | Assessment | Auscultated or counted breaths (among children with history of cough) | Included |
| 0.85 | Assessment | Assessed history of diarrhea | Included |
| 0.85 | Assessment | Assessed history of severe vomiting during illness | Included |
| 0.825 | Assessment | Assessed history of convulsions with illness | Included |
| 0.775 | Assessment | Skin turgor assessed (among children with history of diarrhea) | Included |
| 0.65 | Assessment | Auscultated or counted breaths (among children with history of cough) | Included |
| 0.65 | Assessment | Checked for neck stiffness (among children with history of fever) | Included |
| 0.625 | Assessment | Asked about feeding / breastfeeding habits during illness | Included |
| 0.625 | Assessment | Child weighed | Included |
| 0.6 | Assessment | Child’s temperature checked | Included |
| 0.5 | Assessment | Child undressed | Included |
| 0.475 | Assessment | Felt the child for fever or body hotness |  |
| 0.45 | Assessment | Checked for edema | Included for consistency |
| 0.45 | Assessment | Plotted weight on growth chart |  |
| 0.45 | Assessment | Assessed history of ear pain | Included for consistency |
| 0.4 | Assessment | Observed whether child could drink or breastfeed | Included for consistency |
| 0.375 | Assessment | Looked into child's mouth |  |
| 0.375 | Assessment | Looked in child’s ear |  |
| 0.325 | Assessment | Checked for palmar pallor | Included for consistency |
| 0.3 | Assessment | Checked for pallor by looking at conjunctiva |  |
| 0.3 | Assessment | Felt behind ear (among children with history of ear pain) | Included for consistency |
| 0.3 | Assessment | Asked about mother's HIV status |  |
| 0.3 | Assessment | Asked whether child experienced 2 or more episodes of diarrhea lasting 14 days or more | |
| 0.25 | Assessment | Checked for enlarged lymph nodes in 2 or more of the following sites |  |
| 0.225 | Assessment | Asked about TB infection in any parent in the last 5 years |  |
| 0.775 | Counsel | Described signs and/or symptoms in the child for which to immediately bring child back | Included |
| 0.725 | Counsel | Told the caretaker to continue feeding the child during this illness | Included |
| 0.725 | Counsel | Caretaker is comfortable or confident about how to administer home medications | Included |
| 0.7 | Counsel | Explained how to administer oral treatment(s) to be taken at home | Included |
| 0.65 | Counsel | Told the caretaker to give extra fluids to the child during this illness | Included |
| 0.65 | Counsel | Discussed follow-up visit for the sick child | Included |
| 0.625 | Counsel | Told the caretaker what illness(es) the child has | Included |
| 0.6 | Counsel | Asked the caretaker to repeat the instructions for giving medications at home | Included |
| 0.475 | Counsel | Caretaker is able to discuss problems or concerns with provider |  |
| 0.425 | Counsel | Caretaker satisfied with the amount of explanation received about the problem or treatment |  |
| 0.425 | Counsel | Gave the first dose of the oral treatment |  |
| 0.275 | Counsel | Privacy from having others see the consultation |  |
| 0.275 | Counsel | Privacy from having others hear the consultation |  |
| 0.175 | Counsel | Used a visual aid to educate caretaker |  |
| 0.625 | Integrated care | Looked at the child’s immunization card or asked caretaker about child vaccination history |  |
| 0.525 | Integrated care | Provided general information about feeding or breastfeeding the child even when not sick | Included |
| 0.4 | Integrated care | Mentioned the child’s weight or growth to the caretaker, or discussed growth chart | Included for consistency |
| 0.375 | Integrated care | Asked about normal breastfeeding habits or practices when the child is not ill |  |
| 0.325 | Integrated care | Asked about normal feeding habits or practices when the child is not ill |  |
| 0.325 | Integrated care | Looked at the child’s health card either before beginning the consultation, or while collecting information from the caretaker, or while examining the child |  |
| 0.325 | Integrated care | Asked if child received Vitamin A within past 6 months | Included for consistency |
| 0.325 | Integrated care | Wrote on the child's health card |  |
| 0.225 | Integrated care | Asked if child received any de-worming medication in last 6 months | Included for consistency |
| 0.75 | TXT - Referral | Explained the reason for referral | Included |
| 0.75 | TXT - Referral | Gave pre-referral treatment | Included |
| 0.75 | TXT - Referral | Explained where (or to whom) to go | Included |
| 0.725 | TXT - Referral | Explained when to go for referral | Included |
| 0.675 | TXT - Referral | Gave referral slip to caretaker | Included |
| 0.9 | Treatment | Given antimalarial with diagnosis of malaria | Included |
| 0.825 | Treatment | Given IV with diagnosis of severe dehydration | Included |
| 0.825 | Treatment | Given injectable antibiotic with diagnosis of septicemia | Included |
| 0.825 | Treatment | Given an appropriate antibiotic with diagnosis of pneumonia / bronchopneumonia | Included |
| 0.8 | Treatment | Given injectable antibiotic with diagnosis of meningitis | Included |
| 0.775 | Treatment | Given antibiotic with diagnosis of typhoid fever | Included |
| 0.775 | Treatment | Admitted or referred with diagnosis of severe malnutrition | Included |
| 0.7 | Treatment | Given ORT or IV with diagnosis of moderate or mild dehydration | Included |
| 0.675 | Treatment | Given vitamin A with diagnosis of measles | Included |
| 0.675 | Treatment | Given antibiotic with diagnosis of mastoiditis | Included |
| 0.65 | Treatment | Malaria diagnosis based on RDT or microscopy | Included |
| 0.65 | Treatment | Counselled on feeding with diagnosis of mild or moderate malnutrition | Included |
| 0.625 | Treatment | Given antibiotic with diagnosis of dysentery | Included |
| 0.6 | Treatment | Given an inhaler or bronochodilator with a diagnosis of bronchial spasm / asthma | Included |
| 0.6 | Treatment | Given antibiotic with diagnosis of UTI | Included |
| 0.55 | Treatment | Given zinc with diagnosis of diarrhea | Included |
| 0.45 | Treatment | Health worker derived a diagnosis |  |
| 0.425 | Treatment | Given antibiotic with diagnosis of acute ear infection | Included for consistency |
| 0.375 | Treatment | Given antibiotic with diagnosis of amebiasis | Included for consistency |
| 0.325 | Treatment | Given paracetamol with diagnosis of unclassified fever |  |
| 0.25 | Treatment | Given paracetamol with diagnosis of malaria |  |
| 0.25 | Treatment | Ear wicking performed with diagnosis of chronic ear infection |  |
| 0.2 | Treatment | Given paracetamol with diagnosis of ear infection |  |
| 0.175 | Treatment | Given paracetamol with diagnosis of measles |  |
